# *Dickeya fangzhongdai* was prevalent and caused taro soft rot when coexisting with the *Pectobacterium* complex, with a preference for Araceae plants

**DOI:** 10.3389/fmicb.2024.1431047

**Published:** 2024-06-25

**Authors:** Jingxin Zhang, Dayuan Sun, Huifang Shen, Xiaoming Pu, Pingping Liu, Birun Lin, Qiyun Yang

**Affiliations:** ^1^Plant Protection Research Institute, Guangdong Academy of Agricultural Sciences, Guangzhou, China; ^2^Guangdong Provincial Key Laboratory of High Technology for Plant Protection, Guangzhou, China; ^3^Key Laboratory of Green Prevention and Control on Fruits and Vegetables in South China, Ministry of Agriculture and Rural Affairs, Guangzhou, China

**Keywords:** *Dickeya fangzhongdai*, *Pectobacterium* species complex, coinfection, virulence difference, genomic comparison

## Abstract

Bacterial soft rot caused by coinfection with *Dickeya* spp. and *Pectobacterium* spp. in hosts can cause successive changes in fields, and it is difficult to prevent the spread of and control the infection. *Pectobacterium* spp. are prevalent in the growing areas of tuberous crops, including taro and potato. Recently, *Dickeya fangzhongdai* has emerged as a virulent pathogen in taro. To determine the prevalence status of the causal agents and evaluate the potential spreading risks of *D. fangzhongdai*, screening and taxonomic classification were performed on phytopathogenic bacteria collected from different taro-growing areas in Guangdong Province, China, and biological and genomic characteristics were further compared among typical strains from all defined species. The causative agents were verified to be phytobacterial strains of *D. fangzhongdai, Pectobacterium aroidearum* and *Pectobacterium colocasium*. *P. aroidearum* and *P. colocasium* were found to form a complex preferring Araceae plants and show intensive genomic differentiation, indicating their ancestor had adapted to taro a long time prior. Compared with *Pectobacterium* spp., *D. fangzhongdai* was more virulent to taro corms under conditions of exogenous infection and more adaptable at elevated temperatures. *D. fangzhongdai* strains isolated from taro possessed genomic components of additional T4SSs, which were accompanied by additional copies of the *hcp*-*vgrG* genes of the T6SS, and these contributed to the expansion of their genomes. More gene clusters encoding secondary metabolites were found within the *D*. *fangzhongdai* strains than within the *Pectobacterium* complex; interestingly, distinct gene clusters encoding zeamine and arylpolyene were both most similar to those in *D*. *solani* that caused potato soft rot. These comparisons provided genomic evidences for that the newly emerging pathogen was potentially equipped to compete with other pathogens. Diagnostic qPCR verified that *D*. *fangzhongdai* was prevalent in most of the taro-growing areas and coexisted with the *Pectobacterium* complex, while the plants enriching *D*. *fangzhongdai* were frequently symptomatic at developing corms and adjacent pseudostems and caused severe symptoms. Thus, the emerging need for intensive monitoring on *D. fangzhongdai* to prevent it from spreading to other taro-growing areas and to other tuberous crops like potato; the adjustment of control strategies based on different pathopoiesis characteristics is recommended.

## 1 Introduction

Soft rot Pectobacteriaceae (SRP), including bacteria from the genera *Dickeya* and *Pectobacterium*, are among the most important bacterial plant pathogens based on their scientific or economic importance (Mansfield et al., 2012). They have been found to infect a wide range of plants, including Gramineae crops (rice, corn, etc.), tuberous crops (potato, taro, carrot, etc.), fruits (banana, etc.), and ornamental plants (orchids, etc.) (Ma et al., 2007). Phytopathogenic bacteria of these two genera frequently coinfect potato plants and cause severe blackleg and tuber soft rot worldwide (van der Wolf et al., 2017; Ma et al., 2018; Rossmann et al., 2018). More recently, the emergence of the virulent pathogen *Dickeya solani* has affected the potato industry in Europe and raised global concerns (van der Wolf et al., 2014a). Under pressure from both local evolving SRP pathogens and invasive foreign pathogens, verified host plants are at risk of coinfection with *Dickeya* spp. and *Pectobacterium* spp. or evolved pathogens with increased virulence; moreover, the expansion of their host ranges poses a threat to potential hosts.

Taro (*Colocasia esculenta*) is a world widely cultivated aroid plant featured with large starchy edible corms, and is essential for food security, especially in Asia, Africa, and the Pacific islands; in 2016, this crop had a global production value of 4.1 billion dollars (Delude et al., 2022). This crop has been widely grown in the Yangtze River and Pearl River basins in China. In Guangdong Province, Zhangxi taro from Lechang, Shaoguan, and Wengang taro from Huadu, Guangzhou, are the most famous products with geographical indications; these taro products are characterized by large corms and are the best-selling products on the market, with an output value of more than 360,000 yuan/hm^2^. In recent years, bacterial soft rot has seriously affected this industry and is common in different growing areas, with incidences ranging from 3 to 15%. Especially in 2023, the incidences reached 40% at the core production areas in Lechang, Shaoguan. Like potato in China, taro is strongly affected by *Pectobacterium* spp. in different regions, including Guangdong, Guangxi, and Fujian. In 2021, *Dickeya fangzhongdai* was first reported to infect taro in China (Huang et al., 2021). This causative agent is also a major threat to orchids and is widely distributed in most orchid growing areas in Guangdong, China (Zhang et al., 2018). Other important crops, including pear (Tian et al., 2016), banana (Yang et al., 2022), and onion (Ma et al., 2019), are also seriously damaged by this virulent pathogen.

*Dickeya* spp. and *Pectobacterium* spp. were found to occasionally cause soft rot diseases in the same host, such as potato, vegetables, and flowers (Van Gijsegem et al., 2021). For a long time, *P. atrosepticum* and *P. carotovorum* were the major pathogenic agents for soft rot diseases in potato, and subsequently, other pathogens gradually emerged, including *D. dianthicola*, *P. brasiliense*, and *P. wasabiae*; in this process, interactions among the pathogenic bacteria could induce their succession, and the newly emerging pathogenic bacteria might gradually spread to larger territories and then become the dominant population (Gross et al., 1991; Yap et al., 2004; Kim et al., 2009). For the newly emerging pathogen on taro isolated in Zhangxi village of Lechan, Shaoguang, Guangdong (Huang et al., 2021), it was not clear whether *D. fangzhongdai* infected the new host by chance or had been spreading in fields.

In 2022, we again isolated *D. fangzhongdai* in another village (Mawu) of Lechan, as well as another causative agent, *Pectobacterium aroidearum*. However, the spread and prevalence of *D. fangzhongdai* in the taro-growing areas of Guangdong, as well as the possibility that this emerging pathogen could become the dominant pathogenic bacterium in taro fields, require further investigation. In this study, the phylogenetic determination of pathogenic bacteria isolated from different growing areas and the identification of different kinds of pathogens in plants were performed to determine the spreading status of *D. fangzhongdai* in the field, as well as the actual composition of causative agents for taro soft rot in fields. The biological and genomic characteristics of typical *D. fangzhongdai* strains isolated from taro were compared to those of *D. fangzhongdai* strains isolated from other hosts and *Pectobacterium* spp. isolated from taro to determine the potential for changes in dominant pathogens and the subsequent risks. The clarification of the prevalence status and possible succession between *D. fangzhongdai* and previously colonized *Pectobacterium* spp. not only provides a theoretical basis for the complex epidemic patterns most likely caused by pathogen interactions but also draws attention to the emergence of virulent pathogens such as *D. fangzhongdai* and helps in the development of strategies for the prevention of spread. This will also provide a reference for addressing the risks posed by the adaptation of *Dickeya* pathogens in other tuberous crops, for example, for infection in the important staple crop potato which was also reported to be strongly affected by *Pectobacterium* spp. in China (Zhang et al., 2012; Jiang et al., 2019; Cao et al., 2021; Li et al., 2021; Handique et al., 2022).

## 2 Materials and methods

### 2.1 Collection of diseased samples and isolation of phytobacteria

From 2022 to 2023, taro corms with typical soft rot symptoms were collected from 10 different locations in Guangdong Province, including three in Lechang city, one in Ruyuan County, two in Wujiang District, two in Renhua County, one in Huadu District, and one in Longmeng County. Small pieces of tissue were removed from the margins between diseased and healthy areas and subjected to surface sterilization. The sterilized sections were placed onto nutrient agar (3 g/L beef extract, 0.5 g/L yeast extract paste, 5 g/L peptone, and 10 g/L agar, pH 7.0) and cultured for 48 h at 32°C. Colonies were selected for hypersensitivity testing on tobacco and 16S rDNA sequencing, and the SRPs were further subcultured three times.

### 2.2 Identification and phylogenetic analyses of causal agents

Genomic DNA was extracted from overnight bacterial cultures with a TIANamp Bacterial DNA Kit (Tiangen Biotech, Beijing, China) according to the manufacturer’s directions. Portions of the housekeeping genes (*dnaX*, *leuS* and *recA*) of SRP were sequenced according to the protocol provided by Portier et al. (2019). Sequences obtained by the Sanger method were manually checked and aligned, and each truncated sequence of three genes was concatenated to construct a phylogenetic tree using MEGA 11 with the maximum likelihood method and a bootstrapping test of 1,000 replicates.

### 2.3 Pathogenicity on the pseudostem and corm

Plants were grown from taro corms and maintained in a greenhouse. The pseudostems of 9- to 10-week-old plants were directly injected with 200 μL of 10^8^ CFU/mL bacteria at 5 cm above ground level, and the corms near the basal pseudostems were also directly inoculated; 200 μL of sterile demineralized water served as the control. Each inoculation was repeated three times. Symptoms on the whole plant were observed during disease development.

To compare the initial infection ability of different SRPs on the taro corms under conditions that simulated exogenous infection, each pipette tip with 200 μL of 10^8^ CFU/mL bacteria or sterile water was used to lightly stab the upper side of the corm, and the stab locations were subsequently wrapped with parafilm to prevent the pipette tip from falling and to prevent drying and leakage of the bacteria. Five corms were inoculated with each tested SRP strains, and five other corms were used as controls. The inoculated corms were kept in plastic bags to maintain humidity. The soft rot symptoms were observed and recorded after 5 d of inoculation.

### 2.4 Bacterial growth at different temperatures

Typical strains of *D. fangzhongdai*, *P. aroidearum* and *Pectobacterium colocasium* were cultured in LB broth overnight, and the cell densities were adjusted to 10^8^ CFU/mL. Fifteen microliters of each tested strain were added to 15 mL of LB broth in a 50 mL centrifuge tube and cultured at 200 rpm and at different incubator temperatures. Each inoculation was repeated three times. The temperatures were set at three different levels for the growth of the SRP: 16°C, 30°C and 42°C. The growth curves of the tested strains were generated during 36 h of bacterial culture based on the OD_600_.

### 2.5 Genome sequencing

The genomic DNA of *D. fangzhongdai* J21-2, *P. aroidearum* B2-6 and T25-1, and *P. colocasium* MT27-1 was extracted from 2 mL of each bacterial suspension using a TIANamp Bacterial DNA Kit, and the quality of the extracted DNA was detected using a Qubit (Thermo Fisher Scientific, Waltham, MA) and Nanodrop (Thermo Fisher Scientific, Waltham, MA). Qualified genomic DNA was fragmented with G-tubes (Covaris, Woburn, MA, United States) and end-repaired. Repaired DNAs were selected by a Blue Pippin system to construct SMRTbell DNA libraries with fragment sizes >10 kb according to the manufacturer’s specifications (PacBio, CA, United States). A Qubit 2.0 fluorometer (Life Technologies, CA, United States) and a Bioanalyzer 2,100 (Agilent, CA, United States) were used to determine library quality and average fragment sizes, respectively. SMRT sequencing was performed on a Pacific Biosciences Sequel (PacBio) at Genedenovo (Guangzhou, China) according to standard protocols.

Qualified genomic DNA was also sonicated randomly and then end-repaired, A-tailed, and adaptor ligated using the NEBNext^®^ ΜLtra™ DNA Library Prep Kit for Illumina (NEB, MA, United States) according to the manufacturer’s protocol. DNA fragments with lengths of 300–400 bp were enriched by PCR. These PCR products were purified using an AMPure XP system (Beckman Coulter, CA, United States) and sequenced on an Illumina NovaSeq 6,000 sequencer (Illumina CA, United States) using paired-end technology (PE 150).

### 2.6 Genomic assembly and annotation

Continuous long reads obtained from SMRT sequencing were *de novo* assembled using Falcon (version 0.3.0) (Chin et al., 2016). Clean reads generated from the Illumina platform were aligned to the assemblies from SMRT sequencing. Inconsistencies between PacBio assemblies and Illumina reads were corrected to improve the quality of the assembly and determine the final genome sequences using Pilon (version 1.23) (Walker et al., 2014). Genomic assembly of *D. fangzhongdai* J21-2, *P. aroidearum* B2-6 and T25-1, and *P. colocasium* MT27-1 revealed complete genomes of 5,191,363 bp (1024.03-fold genome coverage of qualified sequenced reads, GC content of 56.44%), 4,806,190 bp (595.06-fold, 51.82%), 4,976,432 bp (930.97-fold, 51.86%), and 4,759,205 bp (521.38-fold, 51.77%), respectively. Plasmid extraction and agarose gel electrophoresis indicated these sequenced bacteria did not contain the plasmids, as well as several other *D*. *fangzhongdai* strains isolated from taro and orchid (Supplementary Figure S1).

The NCBI prokaryotic genome annotation pipeline was used to predict open reading frames (ORFs) (Tatusova et al., 2016), and 4,441, 4,099, 4,309, and 4,049 ORFs were predicted within the above-mentioned genomes. Furthermore, gene islands, transposons, and prophages were identified using IslandPath-DIMOB (version 1.0.0) (Bertelli and Brinkman, 2018), TransposonPSI (version 20,100,822), and PHAST (version 2.0) (Fouts, 2006), respectively. Gene function was further predicted through alignment to genes deposited in different databases, including the National Center for Biotechnology Information (NCBI) nonredundant protein sequence (Nr), UniProt/Swiss-Prot, Kyoto Encyclopedia of Genes and Genomes (KEGG), Gene Ontology (GO), and Cluster of Orthologous Groups of proteins (COG) databases.

### 2.7 Genomic comparison

The amino acid sequences among the available genomes were compared by Diamond (version 2.0.7) (Buchfink et al., 2021), and similarity clustering was carried out by OrthoMCL (version 1.4) (Li et al., 2003) to obtain a list of clustered homologous genes. A single copy of a homologous gene was selected for multisequence alignment using MUSCLE (version 3.8.31), and a phylogenetic tree was constructed using IQTREE (version 1.6.3) (Nguyen et al., 2015) based on the concatenated single-copy gene sequences.

Collinearity analysis between genomes was performed on MUMmer (version 3.1) (Kurtz et al., 2004) software, and SyRI (version 1.4) (Goel et al., 2019) was used to confirm the local positional arrangement. Pyani (version 0.2.7) (Pritchard et al., 2016) was further used to calculate the average nucleotide identity (ANI) of the comparison regions of each of the two analyzed genomes, and GGDC 3.0 (Meier-Kolthoff et al., 2022) was applied to evaluate the values of *in silico* DNA–DNA hybridization (isDDH). Generally, an ANI of 0.96 and an isDDH of 0.70 are used as the classification thresholds to distinguish different species. Additional genomic comparisons were performed on specific functional features, such as gene clusters of secondary metabolites predicted by antiSMASH (version 4.1.0) (Blin et al., 2017; Chevrette et al., 2017), secretory proteins predicted by SignalP4.0 (Petersen et al., 2011), transmembrane proteins predicted by TMHMM (version 2.0) (Krogh et al., 2001), and TNSSs (type N secretion systems, I-VII types) retrieved from the KEGG and Nr annotations. The classification of predicted T4SSs were based on the mating-pair formation genes (*mpf*) (Guglielmini et al., 2014).

### 2.8 Detection of *Dickeya fangzhongdai* and *Pectobacterium* spp.

Nine large taro-growing fields, which are distributed in most of the important taro-growing areas in Guangdong Province, were selected for the investigation of soft rot disease. Forty-eight symptomatic or asymptomatic corms were collected and used for DNA extraction. The ground corm tissue was processed with a MiniBEST Plant Genomic DNA Extraction Kit (Takara, Beijing, China) according to the manufacturer’s instructions. Primers and probes specific for *D. fangzhongdai* (Tian et al., 2020) and *Pectobacterium* spp. (Arizala et al., 2022) and 100 ng of DNA were included in the reaction mixture with Premix Ex Taq™ (Probe qPCR) (Takara) at the recommended amounts, and qPCR was performed on a CFX96 real-time PCR instrument (Bio-Rad, United States) in triplicate. The cycling conditions were as follows: initial denaturation, 5 min at 95°C; extension, 40 cycles of 15 s at 95°C and 35 s at 60°C. Fluorescence was quantitated at the end of each extension step.

## 3 Results

### 3.1 Co-occurrence of *Dickeya fangzhongdai* and different *Pectobacterium* species in fields

**Figure 1:**
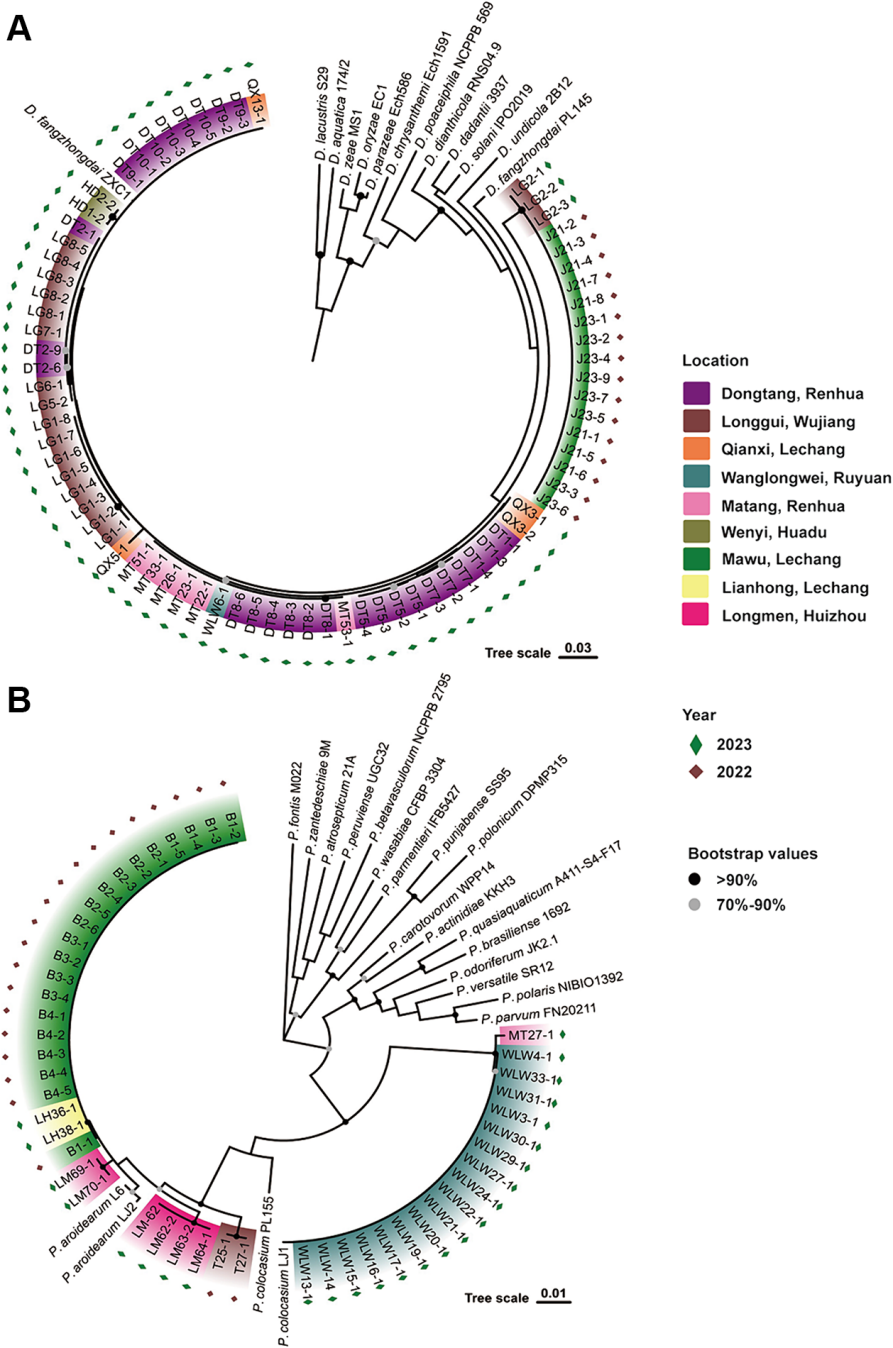
Phylogenetic determination of phytobacteria causing taro soft rot in Guangdong Province, China, from 2022 to 2023. **(A)** *D*. *fangzhongdai* strains along with typical strains of different species in the genus *Dickeya*. **(B)** *P. aroidearum* and *P. colocasium* strains along with typical strains of different species in the genus *Pectobacterium*.

Previously, *D. fangzhongdai* and *Pectobacterium* spp. (*P. aroidearum* and *P. colocasium*) were detected in different fields. In 2022, we first isolated strains of *D. fangzhongdai* (J) and *P. aroidearum* (B) from the same field in Lechan, Shaoguang, and isolated *P. aroidearum* strains (T) from another field in Wujiang, Shaoguang (Figure 1). In 2023, we again sampled diseased taro plants with typical soft rot symptoms from seven different areas of Guangdong Province, China, and isolated 59 strains of *Dickeya* spp. and 26 strains of *Pectobacterium* spp. To determine the actual composition of causative agents for taro soft rot, these pathogenic bacteria were processed to taxonomic classification. Among them, the 59 *Dickeya* strains were all identified as *D. fangzhongdai* according to their close relationship with two *D. fangzhongda*i strains, ZXC1 and PL145, these phylogenetic related strains constructed a clade separating from other *Dickeya* species. The 26 *Pectobacterium* strains were placed into two subgroups, respectively, represented by *P. aroidearum* L6 and LJ2 and *P. colocasium* LJ1, and they were determined as strains from two individual species: *P. aroidearum* and *P. colocasium* (Figure 1). Thus, pathogenic strains of different *Pectobacterium* species occurred in fields, while the pathogens from *Dickeya* genus were validated as the same species, *D. fangzhongdai*. Notably, *D. fangzhongdai* was often isolated from fields where *P. aroidearum* and *P. colocasium* survived.

### 3.2 Biological comparison of *Dickeya fangzhongdai* and *Pectobacterium* spp.

**Figure 2:**
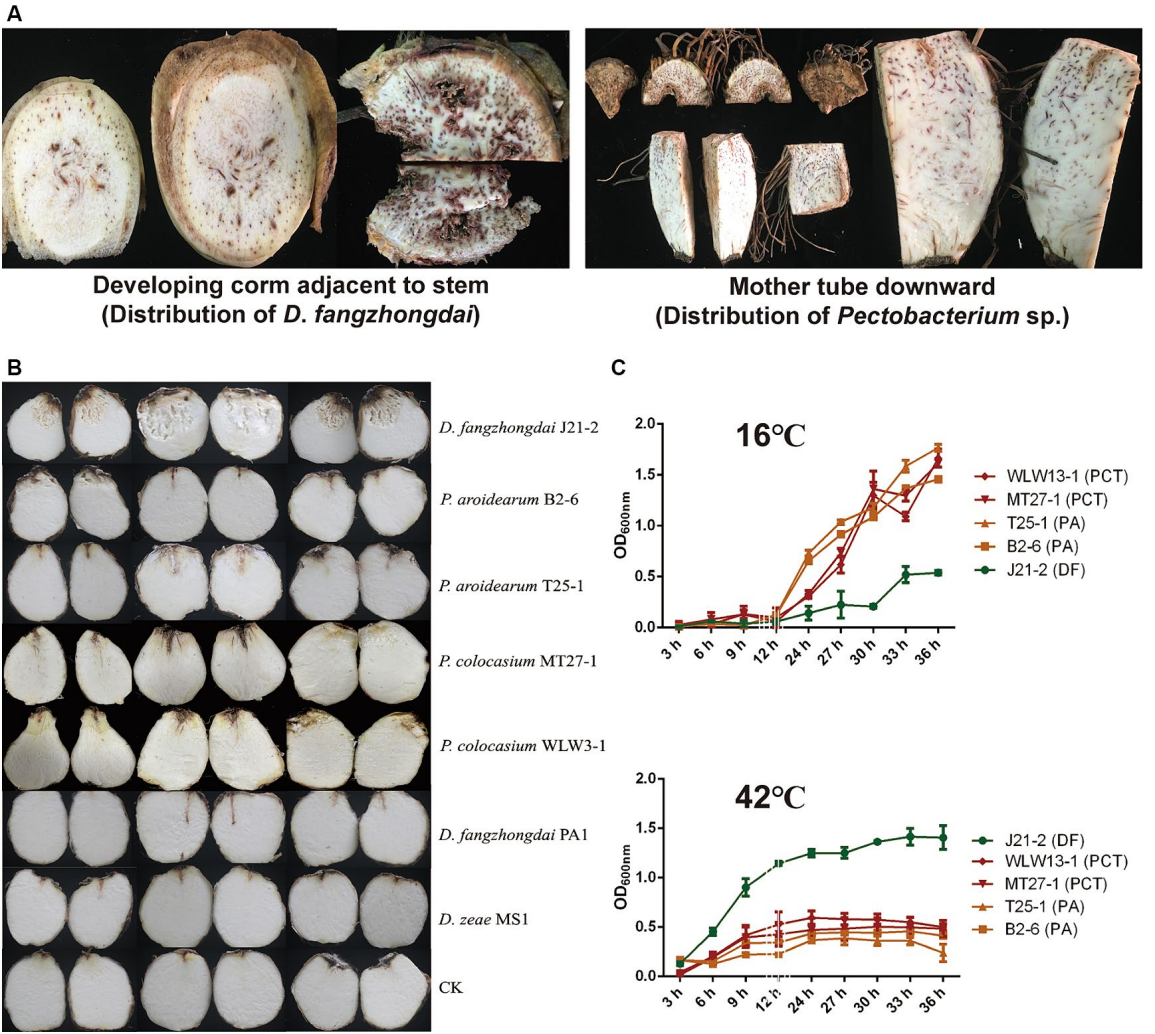
Differences in taro pathogenicity and temperature-dependent growth between *D. fangzhongdai* and *Pectobacterium* spp. **(A)** Symptoms observed in different parts of the same field-collected taro plant and between *D. fangzhongdai* and *P. aroidearum* were only isolated from tissues of developing corms and mother tubers, respectively. **(B)** Differences in virulence between taro corms under conditions of exogenous infection with different SRP strains. **(C)** Differences in growth of strains of *D. fangzhongdai* J21-2, *P. aroidearum* B2-6 and T25-1, and *P. colocasium* MT27-1 and WLW13-1 under different temperatures.

Seed-borne pathogenesis is an important pathogenic mode of taro soft rot, and the typical symptoms of this disease are initiated at the mother tubers. The *D. fangzhongdai* and *P. aroidearum* strains were first isolated from the same plant in 2022 in this study; these strains were isolated from the developing corms near the ground and from the mother tuber downward, respectively. However, typical symptoms were observed only in the corm above rather than in the mother tuber, which was symptom free (Figure 2A), indicating a consequence of exogenous infection. Unlike SRPs, which cause both blackleg in potato stems and soft rot in tubers, the tested strains including *D. fangzhongdai* J21-2, *P. aroidearum* B2-6, and *P. colocasium* MT27-1 could not cause symptoms in the pseudostems of taro, while typical soft rot symptoms developed after direct injection into the interior of the corms, and invasion in the underground part was the only way in which the disease was initiated (Supplementary Figure S2). Under conditions that simulate exogenous infection in corms, *D. fangzhongdai* exhibited a much greater ability to decompose corm tissue than did *Pectobacterium* spp.; similarly, *D. fangzhongdai* presented much greater virulence on taro than did *Dickeya* strains isolated from other hosts, including orchids and banana (Figure 2B). Under favorable conditions for bacterial growth (30°C), both *D. fangzhongdai* and *Pectobacterium* spp. grew well. When they were grown at a lower temperature (16°C), four strains of *P. aroidearum* and *P. colocasium* displayed significantly greater growth density than did the *D. fangzhongdai* strain after 36 h of culture (*p* < 0.01), even though the *D. fangzhongdai* and *Pectobacterium* spp. strains grew slowly during the first 12 h under this nonoptimal condition (Figure 2C). However, they showed opposite growth differences at 42°C; *D. fangzhongdai* adapted well to the elevated temperature and could still grow to moderate bacterial densities in the stationary phase, while *Pectobacterium* spp. developed a much lower population under high-temperature conditions (Figure 2C). Thus, the newly emerging pathogen *D. fangzhongdai* was highly aggressive toward taro and strongly adaptive at elevated temperatures; therefore, this pathogen should receive increased attention.

### 3.3 An Araceae-preferring *Pectobacterium* complex infecting taro

**Figure 3:**
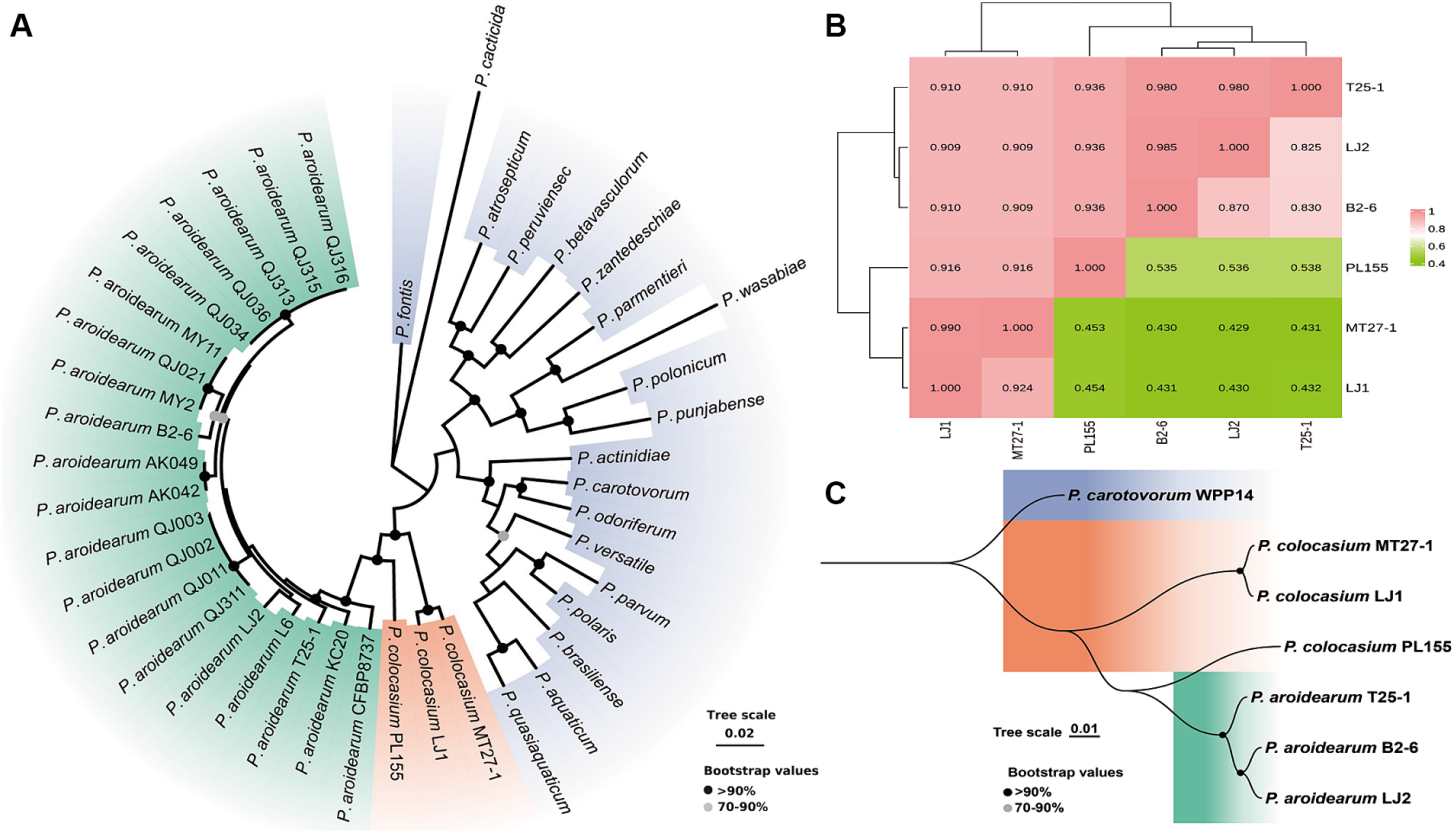
Phylogenetic determination and whole-genome similarities of the Araceae-preferring *Pectobacterium* complex. **(A)** Phylogenetic analyses of typical strains of *P. aroidearum*, *P. colocasium* and 18 other *Pectobacterium* species based on 12 housekeeping genes. **(B)** Heatmap of ANI (numbers on top of the incline) and isDDH values (numbers below the incline) of typical strains of *P. aroidearum* and *P. colocasium*. **(C)** Phylogenetic analyses of typical strains of *P. aroidearum* and *P. colocasium* based on whole-genome sequences.

Validation of the composition of the collected phytobacteria suggested that two different *Pectobacterium* species (*P. aroidearum* and *P. colocasium*) were both important causative agents of the disease (Figure 1). According to phylogenetic analyses of strains from 20 *Pectobacterium* species based on 12 housekeeping genes (*dnaX*, *dnaN*, *gyrB*, *recA*, *acnA*, *pgi*, *proA*, *mdh*, *fusA*, *gapA*, *rplB*, and *rpoS*), typical strains of *P. aroidearum* and *P. colocasium* displayed the closest relationship according to their obvious divergence from 18 other *Pectobacterium* species (Figure 3A), indicating a combination of genetically close pathogens separating other *Pectobacterium* species. Since the strains within *P*. *colocasium* were only recorded on the taro plants and the species was named after the typical host (*Colocasia esculenta*), we further investigate their phylogenetical relationship with the closely related species, *P. aroidearum*. The values of ANI between pairs of *P. aroidearum* B2-6, LJ2, and T25-1 ranged 0.980–0.985 and values of isDDH ranged 0.825–0.870; *P. colocasium* MT27-3 and LJ1 presented 0.990 of ANI and 0.924 of isDDH (Figure 3B). However, the values of ANI and isDDH between *P. colocasium* PL155 from Hawaii and either *P. colocasium* MT27-1 or LJ1 were approximately 0.92 and 0.43, respectively, as indicated the dramatic dissimilarities in this taro-strain-only species (Figure 3B). According to the classification thresholds of different species (ANI: 0.96, isDDH: 0.70), PL155 from Hawaii could not be grouped into *P. colocasium*. However, those values were much lower than those between the PL155 and *P. aroidearum* strains, which were approximately 0.94 and 0.54, respectively (Figure 3B). PL155 presented as an intermediate type linking these two closely related *Pectobacterium* species and was proposed to be separated as a novel species. This was also supported by both phylogenetic analyses based on whole-genome sequences (Figure 3C). Thus, we suggested that these taro pathogens were grouped into a *Pectobacterium* complex consisted of three closely related species, which preferred Araceae plants (Nabhan et al., 2013).

The strong differentiation was not only occurred among *P. colocasium*, but also found among strains *P. aroidearum*. Typical strains of *P. aroidearum* isolated from taro, B2-6 and T25-1, were isolated in the same city but not grouped within a close cluster that could be distinguished from strains isolated from konjak (*Amorphophallus konjac*). Instead, they presented greater genetic distance than that among strains isolated from these different hosts (Supplementary Figure S3). Synteny analyses between B2-6 and T25-1 revealed a genomic arrangement at the majority of the genomic regions, and they presented only moderate similarities: 89.24% for T25-1 and 91.98% for B2-6 (Supplementary Figure S3). Thus, the pathogen infecting taro had differentiated intensively for a long time to develop a complex consisted of varied species, which however were close in phylogenetic relationship.

### 3.4 T4SS Loci contributing to the expansion of *Dickeya fangzhongdai* genomes isolated from taro and additional T6SS effectors

**Figure 4:**
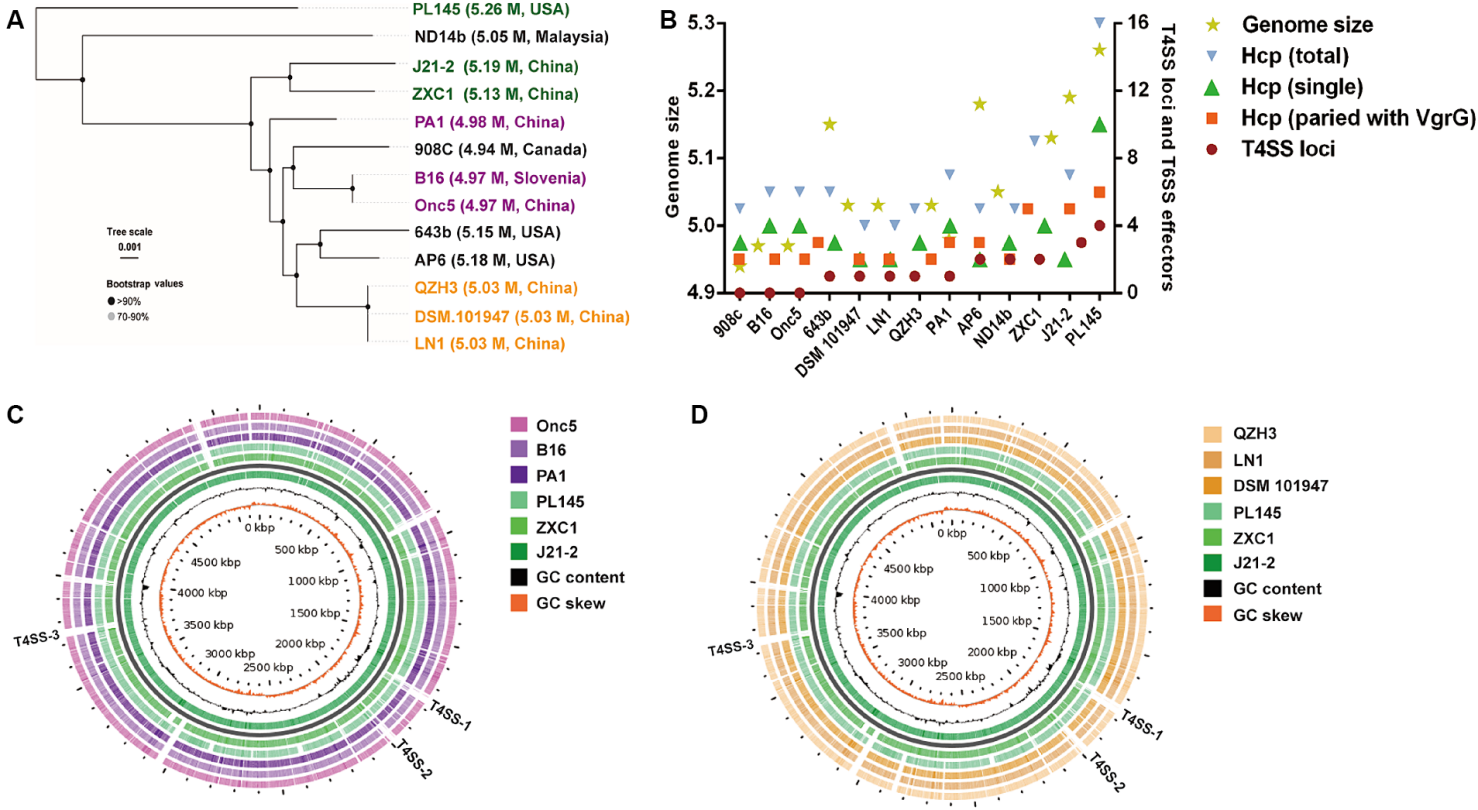
Phylogenetic analysis based on *D. fangzhongdai* genomes and the presentation of typical features for genomic comparison. **(A)** Phylogenetic tree of *D. fangzhongdai* strains isolated from different hosts and different areas. **(B)** Correlations between the number of T4SS loci and genomic size or the number of T6SS effectors (*hcp*, *hcp*-*vgrG*). **(C)** Genome BLAST-Atlas analysis between taro strains and orchid strains. **(D)** Genome BLAST-Atlas analysis between taro strains and pear strains.

Using whole-genome sequences, *D. fangzhongdai* strains from taro (J21-2, ZXC1, and PL145) were distinct from the groups of typical strains isolated from either orchids or pear trees in the phylogenetic analysis (Figure 4A). The genomes of taro strains were much larger than those of the domestic strains from other hosts (Supplementary Table S1). Synteny alignment indicated that 94.99–95.28% of the genomes of orchid strains were aligned to 91.29–91.88% of the J21-2 genome, and 92.85% of the genomic sequences of pear strains were matched to 90.28% of the J21-2 genome (Supplementary Figure S4). Among the *D. fangzhongdai* strains available of genome sequences, specific genes within the strains from taro, J21-2, ZXC1, and PL145, were much greater than the numbers in other *D. fangzhongdai* strains (Supplementary Figure S5; Supplementary Table S2). These results indicated that the taro strains possessed expanded genomes and greatly differed from the domestic strains previously isolated from other hosts.

**Figure 5:**
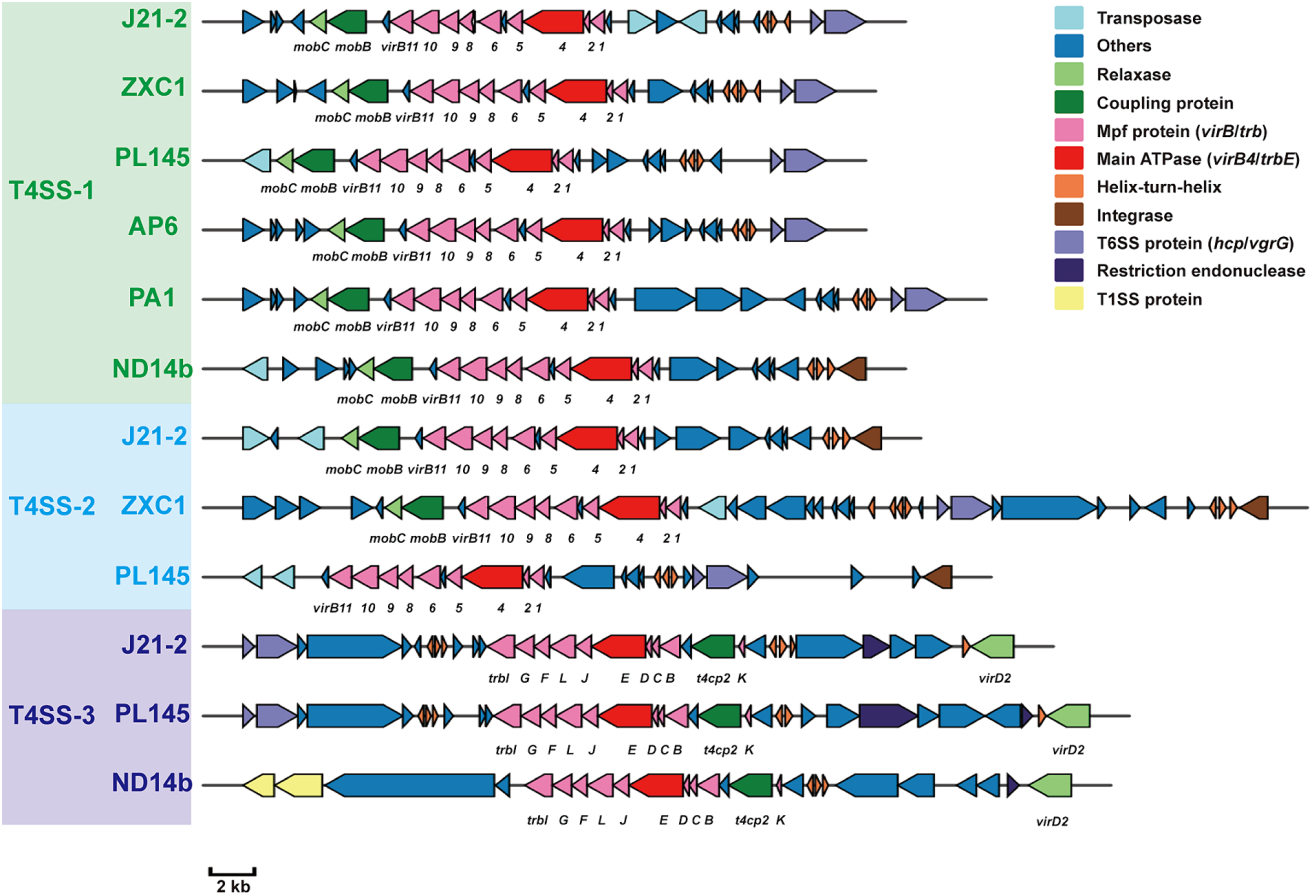
T4SS gene clusters of MPF_T_ family presenting in *D*. *fangzhongdai* strains and their upstream and downstream genomic components. Green text of *D*. *fangzhongdai* strains indicates the strains containing gene clusters within T4SS-1 locus; blue text of *D*. *fangzhongdai* strains indicates the strains containing gene clusters within T4SS-2 locus; purple text of *D*. *fangzhongdai* strains indicates the strains containing gene clusters within T4SS-3 locus. Each gene array informs the constituents of MPF_T_ T4SS and the genomic features at upstream and downstream, including elements of HGT and T6SS. The gene symbols below the gene arrays indicate the typical T4SS genes, including genes encoding Mpf proteins (VirB, Trb), main ATPase (VirB4), relaxases (MobC, VirD2/MobP) and coupling proteins (MobB, T4CP2).

**Figure 6:**
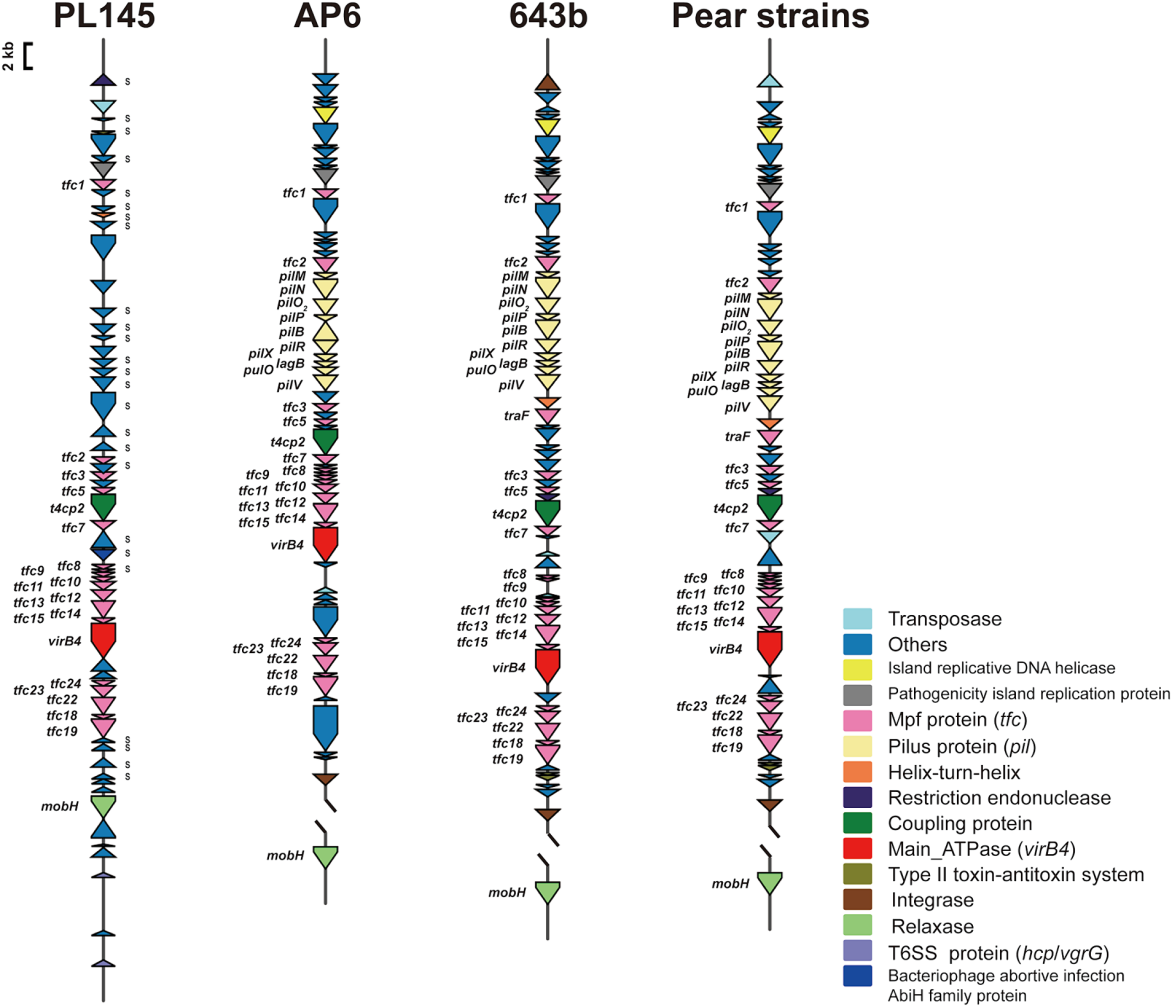
T4SS gene clusters of MPF_G_ family presenting in *D*. *fangzhongdai* strains from taro and pear trees. Each gene array informs the constituents of MPF_G_ T4SS and the genomic features at upstream and downstream, including elements of HGT and T6SS. The gene symbols below the gene arrays indicate the typical T4SS genes, including genes encoding Mpf proteins (Tfc), main ATPase (VirB4), relaxases (MobH) and coupling proteins (T4CP2). S attached to the gene cluster indicates specific genes for strain PL145 from taro. Array of genes encoding Pilus (*pil*) was found between *tfc2* and *tfc3* of MPF_G_ T4SS of strains isolated from pears, as well AP6 and 643b.

Whole-genome comparisons revealed that the number of T4SS loci varied among the *D*. *fangzhongdai* genomes, and the correlation between genome size and the number of T4SS loci was significant (r = 0.8908, *p* < 0.0001) (Figure 4B). BLAST-Atlas analysis revealed that several T4SS gene clusters and accessory genomic components presented in the genomic regions exhibiting large dissimilarities between taro strains and both orchid strains (Figure 4C) and pear strains (Figure 4D). T4SS gene cluster of MPF_T_ family (T4SS-1), *virB11*-*virB1*, was conserved among the taro strains (J21-2, ZXC1 and PL145) and three other strains from other hosts or from environment (PA1, AP6, and ND14b), along with *mobC* encoding relaxase and *mobB* encoding coupling protein (Figure 5). The taro strains had another T4SS gene cluster (*virB11*-*virB1*) of MPF_T_ family at T4SS-2 locus (Figure 5); notably, genes within T4SS-2 locus showed sequence similarities with *Pectobacterium* strains. Additionally, a different T4SS gene cluster belonging to MPF_T_ family, *trbK*-*trbI*, was also found within only taro strains at T4SS-3 locus, including J21-2 and PL145 (Figure 5). These three T4SS clusters of MPF_T_ family were not found in the pear strains, but a T4SS clusters of MPF_G_ family were harbored by these strains; this cluster (*tfc*) was also found among PL145, AP6 and 643b (Figure 6). More interestingly, a ten-gene array encoding type IV pilus proteins were inserted into this T4SS locus, except for the taro strain PL145 which also presented other difference in the genomic characteristics like the adjacent *mobH* gene and many specific genes (Figure 6).

Within this T4SS clusters of MPF_G_ family and its neighborhood genes, PL145 presented a large proportion of specific genes, and 25 of the 61 genes (*PL145_16600*-*PL145_16250*) were not found among the other *D*. *fangzhongdai* strains (Figure 6). Moreover, 11 of these 25 specific genes were most similar to those in *Pectobacterium* strains (Supplementary Table S3). These foreign genomic components were probably acquired by horizontal gene transfer (HGT) from related microorganisms since genes encoding transposase, integrase, pathogenicity island replication protein, and bacteriophage abortive infection AbiH protein were distributed within this T4SS loci (Figure 6). Genomic features related to HGT were also common in the three other kinds of T4SS clusters (Figures 5, 6). In addition to PL145, the taro strain J21-2 had 29 IS transposase and 10 integrase genes among 207 specific genes in comparison with other nontaro strains (Supplementary Table S4), and 26 specific genes were also matched to the sequences of *Pectobacterium* strains, including *P*. *aroidearum* and *P*. *colocasium*. These results indicated that *Pectobacterium* strains are important genomic donors for expanded genomes. Variation in the T4SS cluster mediated by HGT could be an effective tool for acquisition of different foreign DNA fragments and bacterial interactions.

In addition to HGT features, T6SS effectors, such as *hcp* genes or *hcp-vgrG* pair genes, were found to be closely related to T4SS clusters (Figures 5, 6). We found that the number of *hcp*-*vgrG* pair genes was much greater in the taro strains that had the most T4SS loci (correlation r = 0.8397, *p* < 0.001), especially if they had T4SSs of MPF_T_ family; the number of single *hcp* genes was also linked to the T4SS loci (Figure 4B). However, the T4SS of MPF_G_ family did not increase the number of T6SS effectors, and we only detected the *hcp* gene near the T4SS cluster of MPF_G_ family in strain PL145, which does not harbor pilus biogenesis genes (Figure 6).

### 3.5 Distinctive genomic constituents encoding secondary metabolites in *Dickeya fangzhongdai* in comparison with *Pectobacterium* complex

**Figure 7:**
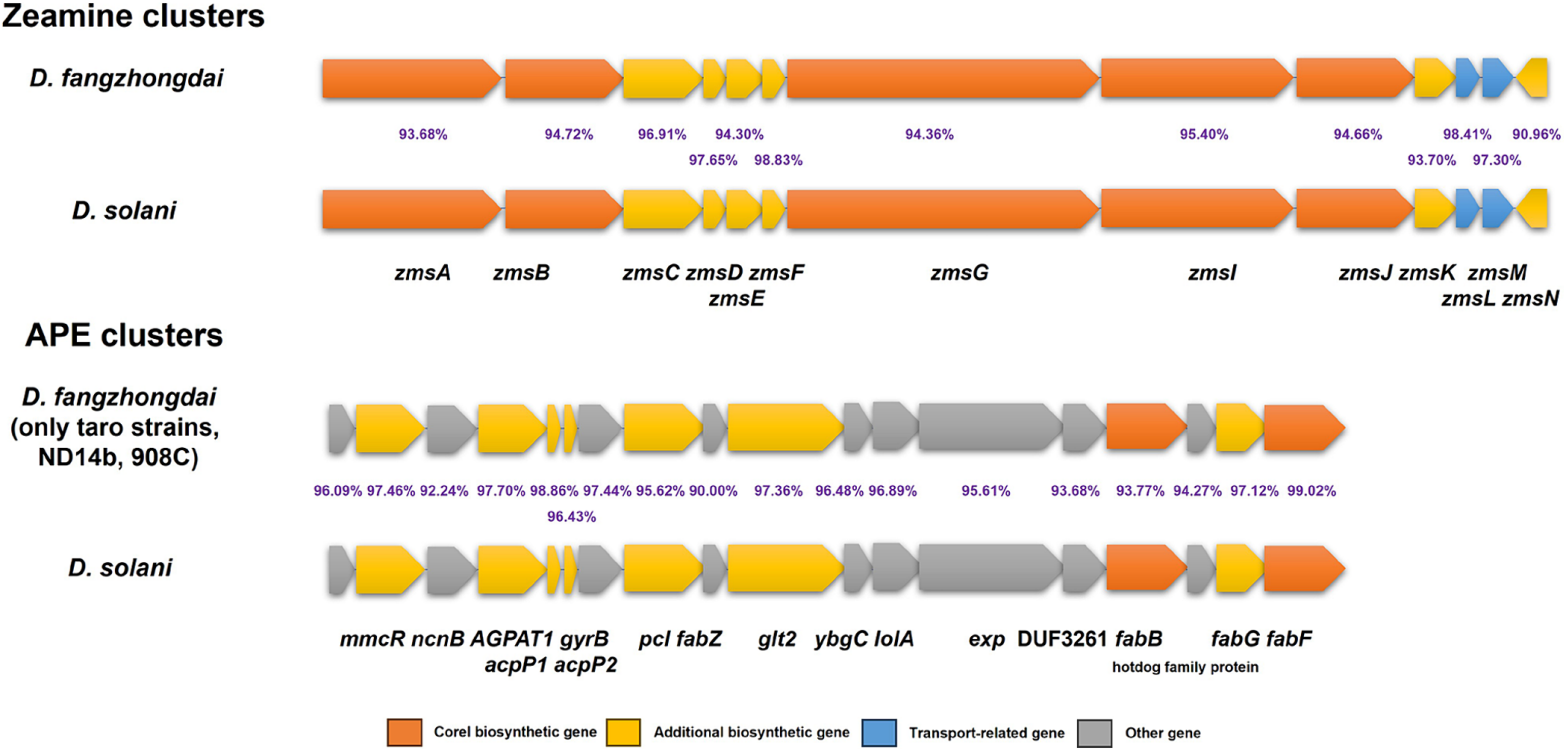
Genomic array of zeamine clusters and arylpolyene (APE) clusters within *D*. *fangzhongdai* and *D*. *solani*. The percentages between each pair of homologous genes indicate the similarities of protein sequences.

According to the nine genomes of three different kinds of taro pathogens, more secondary metabolite biosynthesis-related gene clusters were detected within *D*. *fangzhongdai* strains (10 clusters) than within both *P. aroidearum* strains (6 clusters) and *P. colocasium* strains (5 clusters) by antiSMASH (Supplementary Table S5). Five clusters of *P. colocasium* were all predicted within *P. aroidearum*, and either the siderophore or betalactone cluster was missing in *P. colocasium*. Most of those clusters in *P. aroidearum* and *P. colocasium* were also found among *D*. *fangzhongdai* strains from taro, but the latter harbored other distinct types of secondary metabolite biosynthesis-related gene clusters, such as the zeamine, cyanobactin, and arylpolyene (APE) clusters. Zeamine clusters were conserved among all 13 analyzed *D*. *fangzhongdai* strains, and APE clusters were found only within the taro strains and two other non-host-isolated strains, ND14b and 908C. More interestingly, the zeamine clusters and APE clusters in the *D*. *fangzhongdai* strains were most similar to those in the rising pathogen *Dickeya solani*, which frequently infects the important tuberous crop potato and has high virulence (Figure 7). These indicated that taro strains of *D*. *fangzhongdai* was genetically close to another recently evolved pathogens from tuberous crops at typical genomic features, like zeamine and APE clusters.

### 3.6 Frequent cooccurrence of *Dickeya fangzhongdai* and *Pectobacterium* spp. in fields

The occurrence of taro soft rot was prevalent in the growing areas, soft rot disease was observed in all nine investigated fields in 2023, and 48 symptomatic or asymptomatic corms were collected (Table 1). Using primers and probes specific to *D. fangzhongdai*, 24 corms from eight locations were found to contain this pathogen; using primers and probes specific to *Pectobacterium* spp., a positive reaction was found among 35 corms from eight locations. The newly emerging pathogen *D. fangzhongdai,* as well as *Pectobacterium* spp., is prevalent in the taro-growing areas in Guangdong Province. However, the occurrence of *Pectobacterium* spp. was still more frequent than that of *D. fangzhongdai*. Among the individual taro corms, we found 14 corms coinfected with *D. fangzhongdai* and *Pectobacterium* spp., despite the prevalence of both kinds of pathogens, indicating potential competition in the process of coinfection. Thus, *D. fangzhongdai* was identified as another important causative agent of taro soft rot but still struggled in the presence of *Pectobacterium* spp.

**Table 1:** qPCR detection of *D*. *fangzhongdai* and *Pectobacterium* spp. in taro corms collected from different growing areas.

Location	Samples	*D. fangzhongdai*	*Pectobacterium* spp.	Location	Samples	*D. fangzhongdai*	*Pectobacterium* spp.
Dongtang, Renhua	DT1	✓	×	Longgui, Wujiang	LG1	✓	✓
	DT2	✓	✓		LG2	✓	✓
	DT3	×	✓		LG3	×	✓
	DT4	✓	×		LG4	×	✓
	DT5	✓	✓		LG5	×	✓
	DT6	×	×		LG6	×	✓
	DT7	✓	✓		LG7	×	✓
	DT8	✓	✓		LG8	✓	✓
	DT9	×	✓				
	DT10	×	×				
Matang, Renhua	MT1	×	✓	Dong’an, Ruyuan	DA1	✓	×
	MT2	×	✓		DA2	✓	×
	MT3	✓	✓		DA3	✓	×
	MT4	×	✓		DA4	✓	×
	MT5	×	✓				
	MT6	×	✓				
Lianhong, Lechang	LH1	×	✓	Wanglongwei, Ruyuan	WLW-1	✓	✓
	LH2	×	✓		WLW-2	×	✓
	LH3	✓	✓		WLW-3	×	✓
	LH4	×	✓	Wenyi, Huadu	HD1	✓	✓
Qianxi, Lechang	QX1	✓	×		HD2	✓	✓
	QX2	×	×		HD3	✓	✓
	QX3	✓	✓		HD4	✓	×
	QX4	✓	×	Longmen, Huizhou	LM1	×	✓
	QX5	✓	×		LM2	×	✓
	QX6	×	✓		LM3	×	✓

✓ indicates the positive reaction for the detection of D. fangzhongdai or Pectobacterium spp., and × indicates the negative reaction. Light gray indicates the positive reaction for the detection of D. fangzhongdai, and dark gray indicates the positive reaction for the detection of Pectobacterium spp.

**Figure 8:**
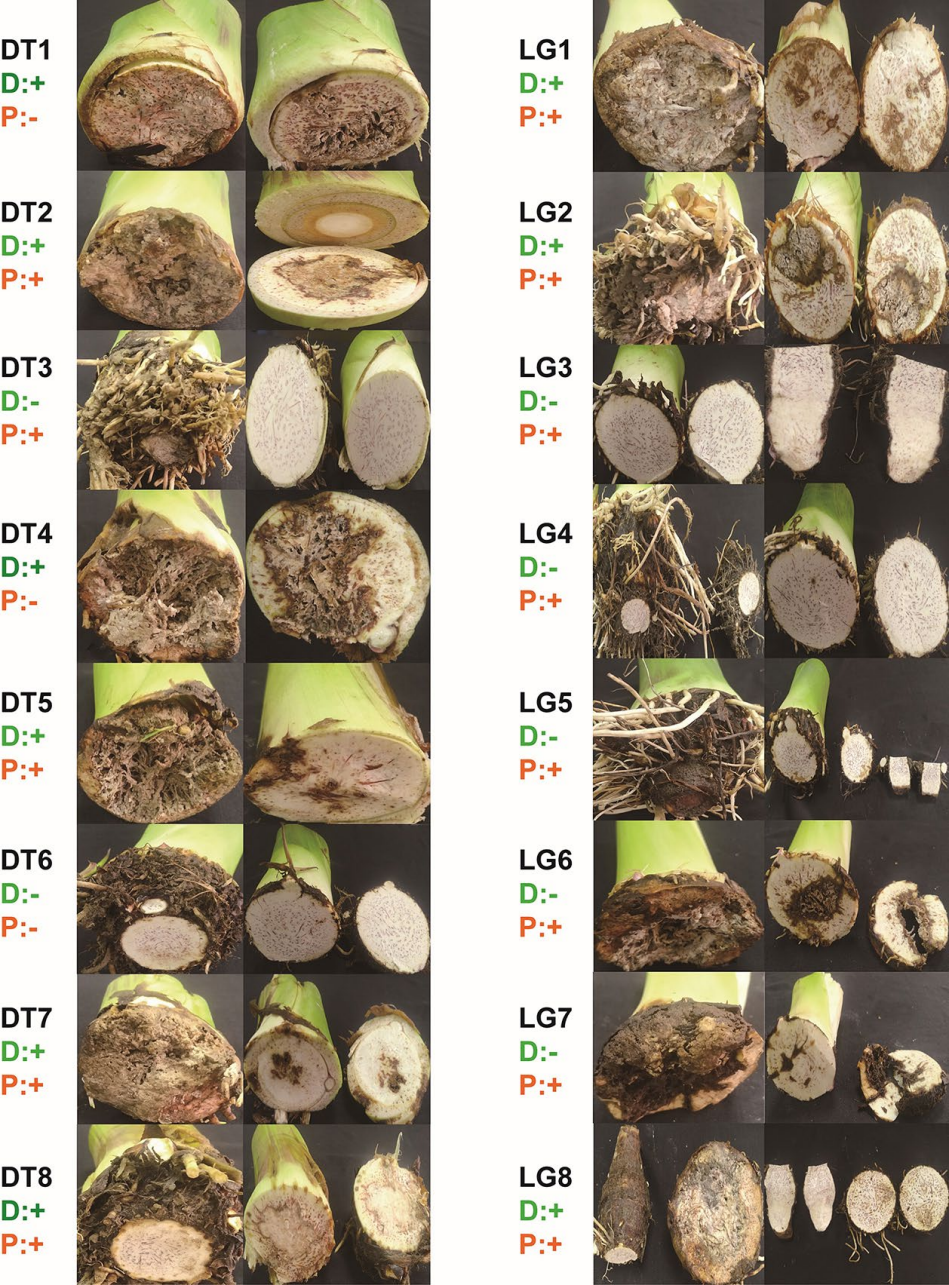
Symptoms on developing corms and mother tubes of taro plants collected from Renhua and Wujiang, Shaoguan city, in June 2023. DT indicates Dongtang in Renhua, LG indicates Longgui in Wujiang, D indicates detection of *D. fangzhongdai* pathogens using qPCR, and P indicates detection of *Pectobacterium* spp. using qPCR. + and – indicate positive and negative, respectively.

Nevertheless, *D. fangzhongdai* caused soft rot symptoms at the developing corms and their adjacent basal pseudostems more frequently, rather than *Pectobacterium* spp. Comparison of typical symptoms appearing on the samples collected from Dongtang, Renhua and Longgui, Wujiang at June, 2023, revealed that plants enriching *D*. *fangzhongdai* showed severe symptoms at the developing corms adjacent to basal pseudostems with or without the presence of *Pectobacterium* spp., and the symptoms developed up to the basal pseudostems (Figure 8). In contrast, plants with *Pectobacterium* spp. were always found to be symptom-free at the developing corms and their adjacent pseudostems in the absence of *D*. *fangzhongdai* (Figure 8). These were consistent with the observation of their virulence difference under conditions of exogenous infection.

## 4 Discussion

### 4.1 Adaption at elevated temperatures and virulence under exogenous infection raised public attention on *Dickeya fangzhongdai*

Soilborne bacterial diseases seriously affect the growth of various crops, and their causative agents could be combinations of different pathogenic bacteria. In many cases, these pathogens, including SRP pathogens, were found to simultaneously infect the same plant (Fitt et al., 2006). During the occurrence and epidemic spread of SRP, the increase in temperature is an essential driver of the colonization and propagation of emerging pathogens (Degefu et al., 2013; Potrykus et al., 2016). According to the Blue Book Climate Change in China 2023, the period from 2015 to 2022 was the warmest eight-year period on record, and the global average temperature in 2013–2022 increased by 0.05°C compared with that in 2011–2020. Previously, taro soft rot was usually observed in June in Guangdong Province, but the onset period of this disease has shifted to early May in recent years. We further found that the maximum temperatures in May in Shaoguan city, Guangdong, presented an increasing trend (Supplementary Figure S6). The newly emerging causative agent *D*. *fangzhongdai* grew much better at elevated temperatures than did *Pectobacterium* spp. (Figure 2C), indicating that *D*. *fangzhongdai* could be more adaptable to warmer climates. The increasing temperatures could drive the succession of these different pectinolytic bacteria and increase the possibility of *D*. *fangzhongdai* being a dominant phytobacterium in the field (Toth et al., 2011).

In addition to the seed-borne mode, exogenous infection and related virulence differences among different pathogens could be additional concerns associated with this emerging pathogen. Under simulated conditions of exogenous infection, *D*. *fangzhongdai* had a much greater ability to decompose corm tissue than did *Pectobacterium* spp. and caused typical soft rot symptoms with a soaked appearance (Figure 2B). This difference was consistent with the observations of plant samples collected in different fields, as supported that *D*. *fangzhongdai* was more likely to invade externally and cause symptoms on developing corms (Figure 8). In contrast, *Pectobacterium* spp. more commonly caused infection via the seed-borne mode than did *D*. *fangzhongdai*, but the *Pectobacterium*-positive mother tubers sometimes remained symptomless (Figure 8). Thus, caution should be taken when using *D. fangzhongdai* for treatment in the taro industry because of the frequency of exogenous infection and its ability to cause initial tissue breakage. Similarly, *D*. *solani* was more infectious and adaptable in Europe than were previously colonized *Pectobacterium* spp., and it was able to cause symptoms under conditions of lower bacterial density (van der Wolf et al., 2014b). *D*. *solani* has become the dominant pathogenic bacterium causing potato soft rot in Europe. *D*. *fangzhongdai* also causes great losses in the taro industry when present with *Pectobacterium* spp., and therefore, whether *D*. *fangzhongdai* has become the dominant phytobacterium and spread more widely should be evaluated.

### 4.2 *Pectobacterium* complex provided evidence for the existence of “indigenous” population and *Dickeya fangzhongdai* should have interacted with them in pursuit of colonization

SRP pathogens can survive in the reproductive organs or residues in plant soil and surface water and become “indigenous” pathogenic bacteria in local agroecosystems after long-term colonization (Czajkowski et al., 2010, 2011; Ge et al., 2021). In this study, we found typical strains of *P. aroidearum* and *P. colocasium* were closest in phylogenetic relationship, but they showed dramatic differentiation in each species. First, *P. aroidearum* strains have been found on many hosts, but most of them were reported to infect Araceae plants, including taro, konjak, *Syngonium podophyllum*, *Pinellia ternate*, and *Zantedeschia aethiopica* (genomic data from NCBI; Nabhan et al., 2013; Zhou et al., 2022). In particular, in China, *P. aroidearum* has prevailed in the growth areas of different Araceae tuberous crops, such as taro and konjak, and the genomic dissimilarities among strains from taro were even larger than those across different hosts. Second, the strains from taro-exclusive species, *P*. *colocasium*, varied greater in genomic similarities. PL155 from Hawaii should be classified into a novel species based on the species thresholds (ANI: 0.96, isDDH: 0.70), and the novel species was proposed as *Pectobacterium hawaiiensis* sp. nov. The dramatic genomic differentiation occurred among those causative agents of taro soft rot which were assigned to three species in a complex preferring Araceae plants, it was thus indicated that the ancestor of this *Pectobacterium* complex had adapted to the taro host for quite a long time and was thus can be considered “indigenous” pathogenic bacteria in taro fields. On the other hand, newly emerging pathogenic agents are considered foreign invaders and interact with the “indigenous” population in pursuit of colonization, as will determine the ability to geographically spread and the possibility of population dominance.

*D. dianthicola* was once the prevailing causative agent of soft rot in potato in Europe, and subsequently, *D. solani* emerged in the field; this foreign pathogen successfully colonized and coexisted with *D. dianthicola* through competitive interactions (Blin et al., 2021). This situation is common among SRP pathogens coinfecting the same host in the same region (Ge et al., 2021; Motyka-Pomagruk et al., 2021). In this study, *D*. *fangzhongdai* would also compete with the *Pectobacterium* complex in the process of infection onset and field colonization for wide-spreading, since they shared similar ecological niches and caused similar symptoms in the host (Czajkowski et al., 2011). However, stronger virulence did not imply that the pathogens were more competitive and would always dominate among coexisting pathogenic populations. The virulence of *D. dianthicola* was greater than that of *P. parmentieri* coexisting in the same region, while *D. dianthicola* was not able to suppress its competitor, and it became the minor pathogenic bacterium. The interactions of different pathogens infecting the same host are complicated, and their succession is not simply determined by differences in virulence. The genomic diversity and typical genomic features of different coexisting SRP pathogens have previously been found to be correlated with their prevalence and virulence (Yap et al., 2004; Kim et al., 2009), and in this study, the genomic comparisons of different kinds of coexisting SRP strains might provide a feasible way to evaluate the prevalence of *D*. *fangzhongdai*.

Genomic comparison of available genomes of *D*. *fangzhongdai* strains isolated from different hosts and different areas revealed that the acquisition of multiplex T4SSs via HGT increased the genome sizes of the taro strains (Figure 4). As an exceptionally versatile system, the T4SS mediates the transfer of conjugative DNA and protein substrates to recipient cells and DNA uptake from or release into the extracellular milieu (Fronzes et al., 2009) and is necessary for bacterial survival, host-pathogen interactions, and biotic and abiotic adhesion (Costa et al., 2024). The distinctive T4SS and the portable pilus cluster were found to reflect the motility, virulence and biofilm formation of *D*. *fangzhongdai* isolated from pear trees and might contribute to its adaptation to woody hosts (Chen et al., 2020). Compared with the typical hosts reported, taro could be considered a distinctive type of host since its vegetative reproduction occurs via the formation of tuberous organs abundant in starch, protein, crude fiber, and polysaccharides, and it was initially infected only in the underground part rather than in the pseudostem (Supplementary Figure S1). Diversification of the T4SS could also have assisted *D*. *fangzhongdai* in colonizing a distinctive type of host and introducing more genomic components. Like taro strain-specific T4SS-2 arrays, specific genes that were most similar to those for *Pectobacterium* strains were well distributed within the genomes of taro strains in comparison with non-taro strains in *D*. *fangzhongdai*. The closely related genus *Pectobacterium* were considered to be the important genomic donors for the delivery of DNA fragments to *D*. *fangzhongdai*. These provided evidence that primary infection and successful colonization by *D*. *fangzhongdai* involved bacterial interactions with *Pectobacterium* spp.

### 4.3 Genomic comparison revealed potential competitiveness of *Dickeya fangzhongdai* in bacterial interaction

Unlike the interactions between phytopathogenic bacteria and host plants, the relationships among pathogenic bacteria coexisting within the same ecological niches have rarely been elucidated (Xin et al., 2018). Competition and cooperation are the main ways in which these pathogenic bacteria interact (Abdullah et al., 2017), and competition is more common than cooperation (Bauer et al., 2018). The production of antimicrobial compounds, contact-dependent growth inhibition (CDI), and quorum sensing are the most intensively studied mechanisms of bacterial competition (Hibbing et al., 2010). Interestingly, we found that the T4SS gene cluster of MPF_T_ always carried T6SS effectors and obviously increased the number of copies of *hcp*-*vgrG* pair genes among *D*. *fangzhongdai* strains from taro. The T6SS provides an intercellular transport pathway for contact-dependent effectors and mediates bacterial interactions (Schwarz et al., 2010). Hcp and VgrG are substrate and structural components of the T6SS and are structurally related to the T4 bacteriophage tail proteins (Galan and Waksman, 2018); with the aid of this protein pair, various other active toxin effectors are delivered to neighbors directly to contribute to bacterial competition (Russell et al., 2014). For example, the recombination hotspot protein (Rhs) effector in *D. dadantii* exhibited nuclease activity at the C-terminal toxic domain and was proven to inhibit the growth of adjacent cells (Koskiniemi et al., 2013). A more diverse arsenal of Hcp-VgrG in *D*. *fangzhongdai* strains from taro could outperform competitors since they are more likely to transfer distinctive toxin effectors.

As a method of noncontact-dependent growth inhibition, the production of antimicrobial compounds to mediate competition between different species or between related strains of the same species has been verified (Hibbing et al., 2010). Bacteria produce arrays of antimicrobial nonribosomally synthesized peptides (NRPs) and polyketides (PKs), which are typically encoded by adjacent genes. Gene clusters encoding NRPSs and PKSs are common in the SRP genomes, and some of these genes produce metabolites with antibacterial activity (Minowa et al., 2007). Various bacteriocins (carbapenem, carocins, pectocin, pyocin, and carotovoricin) are secreted by *Pectobacterium* spp. and have been reported to directly inhibit a range of bacteria, including closely related bacteria in both the *Pectobacterium* genus and *Dickeya* genus (Van Gijsegem et al., 2021). However, genes encoding these bacteriocins were not predicted among the taro strains of the *Pectobacterium* complex, while gene arrays encoding other types of NRPSs and PKSs were relatively conserved (Supplementary Table S5). Instead, many more secondary metabolite biosynthesis gene clusters were predicted within *D*. *fangzhongdai* from taro, including the zeamine cluster, cyanobactin cluster and APE cluster. In the genus *Dickeya*, this zeamine cluster was first verified to be involved in the virulence of *D. zeae* strains from rice (Zhou et al., 2015) and is mainly distributed within *D. zeae*, *D*. *fangzhongdai,* and *D. solani*. The sequences of the zeamine clusters within *D*. *fangzhongdai* were most similar to those within *D. solani*, and the high GC content (≈61%) suggested that these clusters might have been acquired by HGT (Zhang et al., 2018). The zeamine clusters within *D*. *fangzhongdai* shared high similarities with those within *D. solani*, which might indicate a potential function of competitiveness in the infection onset on the tuberous crops, since *D. solani* has been documented to compete with previously colonized SRP pathogens when they were coinfecting potato plants. More interestingly, the APE clusters within the taro strains were also most similar to those in *D. solani*, while there was no homolog within *D*. *fangzhongdai* strains from other plants. APE pigments have been found to protect bacteria against oxidative stress and assist them in adapting to the surrounding environment (Grammbitter et al., 2019; Johnston et al., 2021). Thus, the production of secondary metabolites such as zeamine and APE might enhance the competitiveness and adaptation to typical crops and relevant surroundings.

### 4.4 *Dickeya fangzhongdai* coexisted with the *Pectobacterium* complex in fields and their successive changes were ongoing

In our experience, it was more difficult to isolate *Pectobacterium* pathogens from diseased samples than to isolate *D*. *fangzhongdai*, especially from samples that were decayed seriously and were parasitized with plenty of saprophytic bacteria, and *Pectobacterium* pathogens were often not obtained in subcultures since the chosen colonies always contained mixtures of endophytic and environmental bacteria, which could indicate relative competitive weakness in the *Pectobacterium* complex. Genomic comparison also provided insight into the possible competitive advantage of the newly emerging pathogen *D*. *fangzhongdai* in comparison with the “indigenous” population of *Pectobacterium* spp. Nevertheless, the composition of the pathogenic bacterial population and relative proportions of members were determined not only via evolutionary gains but also via the response to environmental signals. Different pathogens might form a stable community over the long term. In this study, both *D. fangzhongdai* and *Pectobacterium* spp. were prevalent in different important taro-growing areas in Guangdong Province, and *Pectobacterium* spp. were still common (Table 1). In northern Finland, *D*. *solani* was also found to coexist with four different *Pectobacterium* species, and *Pectobacterium* species also appeared to be more frequent (Degefu, 2021). It is highly likely that *D. fangzhongdai* and *Pectobacterium* spp. can coexist in fields for long periods, and the spread of *D. fangzhongdai* should be monitored and prevented because of its particularity in terms of environmental adaptation, invasion versatility, and evolved arsenal for bacterial interaction. In addition to the use of clean mother tubers, control procedures should be performed earlier, as should preventive measures to reduce external injury and cross-infection. Notably, our pathogen surveillance on potato in early 2024 indicated that *P. aroidearum* occurred in the fields of Enping City, Guangdong with previously reported causative agents of potato black leg, *P. carotovorum* (Zhang et al., 2012) and *P. brasiliense* (Jiang et al., 2019) (data not shown), suggesting a chance of spread of pathogenic bacteria from taro to potato. Thus, the risks of spread to potato cannot be ignored, and this study will also mitigate the risks posed by *D. fangzhongdai.*

## 5 Conclusion

In this study, actual composition of causative agents of taro soft rot were identified to be different SRP bacterial species, including *D. fangzhongdai, P. aroidearum*, and *P. colocasium*. The typical *Pectobacterium* strains varied greatly, exhibiting both intra- and interspecies differences, but they were still phylogenetically close and were considered to cluster into a typical species complex. Compared with these previous colonizing pathogens, *D. fangzhongdai* emerged later and displayed greater virulence on taro corms under conditions of exogenous infection and better growth at elevated temperature, warranting urgent attention as it could frequently set on developing corms and cause greater loss in fields. Through HGT, *D. fangzhongdai* strains from taro acquired genomic components of additional T4SSs accompanied by *hcp*-*vgrG* genes of the T6SS, which contributed to the expansion of the genome. Among the secondary metabolite biosynthesis gene clusters distinct from those in the *Pectobacterium* complex, the zeamine and arylpolyene clusters in the *D. fangzhongdai* strains were both most similar to those in *D*. *solani*. Benefiting from these genomic components, the *D. fangzhongdai* strains could be equipped to compete with the *Pectobacterium* complex in the field. Accordingly, detection in field samples indicated that *D. fangzhongdai* prevailed in most of the taro-growing areas in Guangdong Province and coexisted with the *Pectobacterium* complex. Thus, the newly emerging pathogen *D. fangzhongdai* requires close attention in different taro-growing areas and in different tuberous crops.

## Data availability statement

Publicly available datasets were analyzed in this study. This data can be found here: the sequencing read data of strains of *D. fangzhongdai* J21-2, *P. aroidearum* B2-6 and T25-1, and *P. colocasium* MT27-1 are under the BioProject accession no. PRJNA890429 / Sample accession no. SAMN31278287, PRJNA890439 / SAMN31278450, PRJNA890452 / SAMN31278767, PRJNA1050810 / SAMN38754036, respectively. Genome assembly and annotation strains of *D. fangzhongdai* J21-2, *P. aroidearum* B2-6 and T25-1, and *P. colocasium* MT27-1 were deposited in NCBI GenBank under accession nos. CP109768, CP109769, CP109770, and CP140126, respectively.

## Author contributions

JZ: Writing – review & editing, Writing – original draft, Visualization, Validation, Supervision, Resources, Project administration, Methodology, Investigation, Funding acquisition, Formal analysis, Data curation, Conceptualization. DS: Writing – review & editing, Writing – original draft, Visualization, Validation, Methodology, Investigation, Formal analysis, Data curation. HS: Writing – review & editing, Writing – original draft, Visualization, Validation, Methodology, Investigation, Formal analysis, Data curation. XP: Writing – review & editing, Writing – original draft, Visualization, Methodology, Investigation, Formal analysis, Data curation. PL: Writing – review & editing, Writing – original draft, Visualization, Validation, Methodology, Investigation, Formal analysis, Data curation. BL: Writing – review & editing, Writing – original draft, Supervision, Project administration, Investigation, Funding acquisition, Conceptualization. QY: Writing – review & editing, Writing – original draft, Visualization, Validation, Supervision, Resources, Project administration, Investigation, Funding acquisition, Formal analysis, Data curation, Conceptualization.
