# Supplementary material for: Dickeya fangzhongdai was prevalent and caused taro soft rot when coexisting with the Pectobacterium complex, with a preference for Araceae plants
Source: Front Microbiol. 2024 Jun 25;15:1431047. doi: 10.3389/fmicb.2024.1431047 (PMC11231085; doi:10.3389/fmicb.2024.1431047)
Supplement: Supplementary file 2 [file Data_Sheet_1.PDF]

Supplementary Figure 1 Agarose gel electrophoresis of plasmid extraction of the whole genome sequenced bacterial strains and other *D. fangzhongdai* strains isolated from taro and orchid.

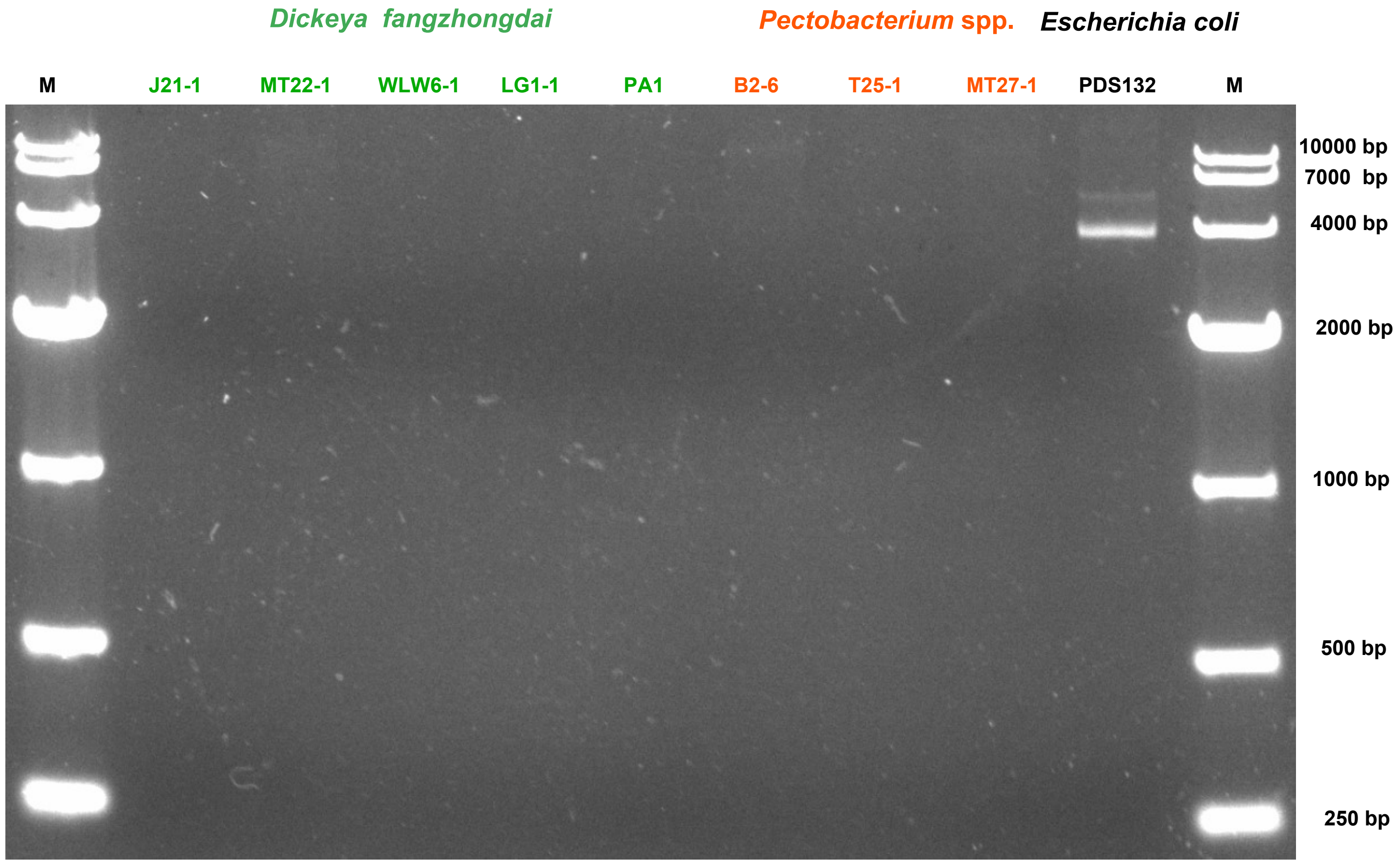
Dickeya fangzhongdai
Pectobacterium spp.
Escherichia coli
M
J21-1
MT22-1
WLW6-1
LG1-1
PA1
B2-6
T25-1
MT27-1
PDS132
M
10000 bp
7000 bp
4000 bp
2000 bp
1000 bp
500 bp
250 bp

Supplementary Figure 2
Disease symptoms caused by direct injection of typical strains of *D. fangzhongdai* and *Pectobacterium* spp. into the pseudostems and corms of taro.

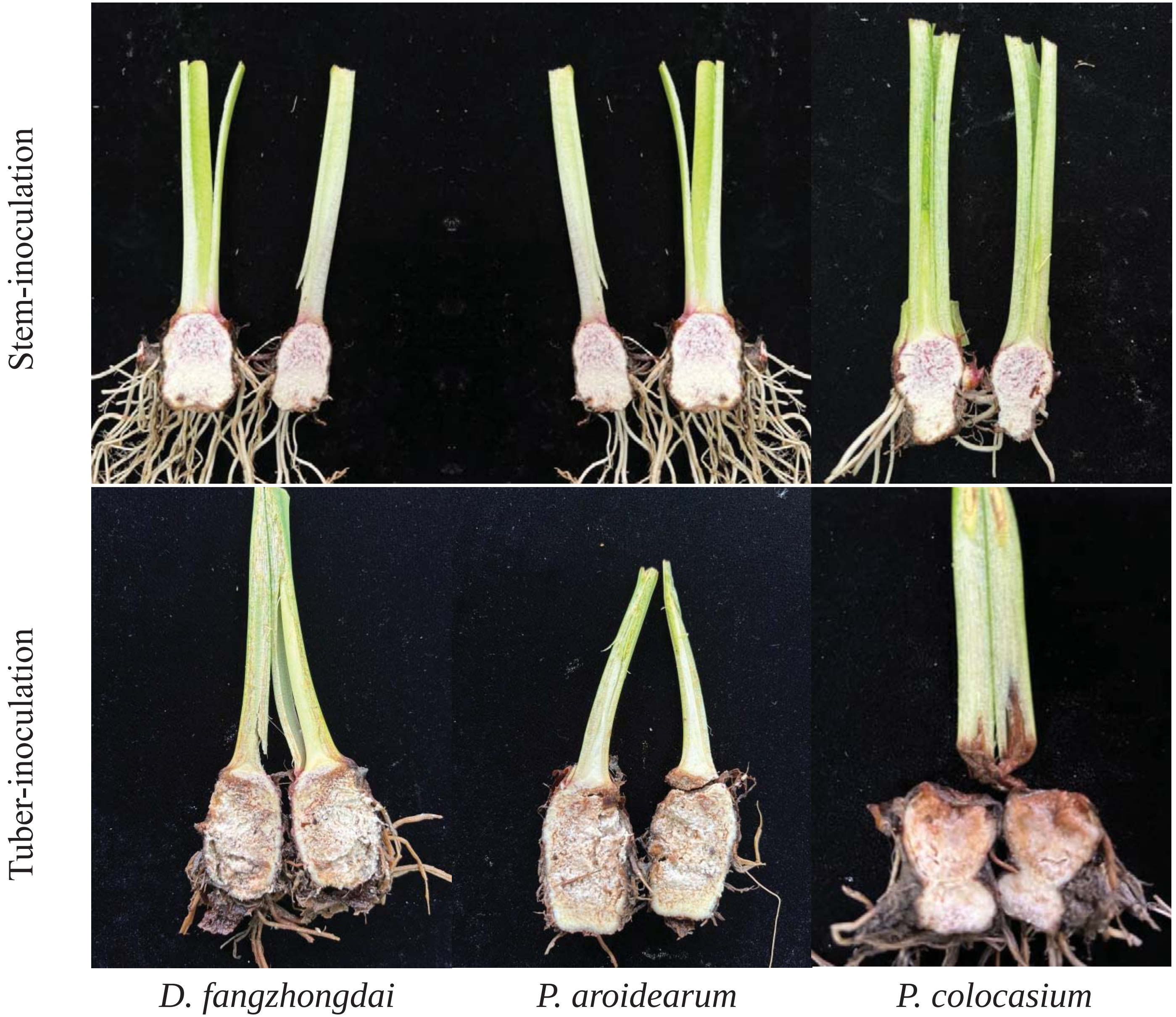
Stem-inoculation
Tuber-inoculation
D. fangzhongdai
P. aroidearum
P. colocasium

Supplementary Figure 3
Heatmap of ANI values between each pair of *P. aroidearum* strains available for whole-genome sequence and synteny alignment of taro strains (B2-6 and T25-1).

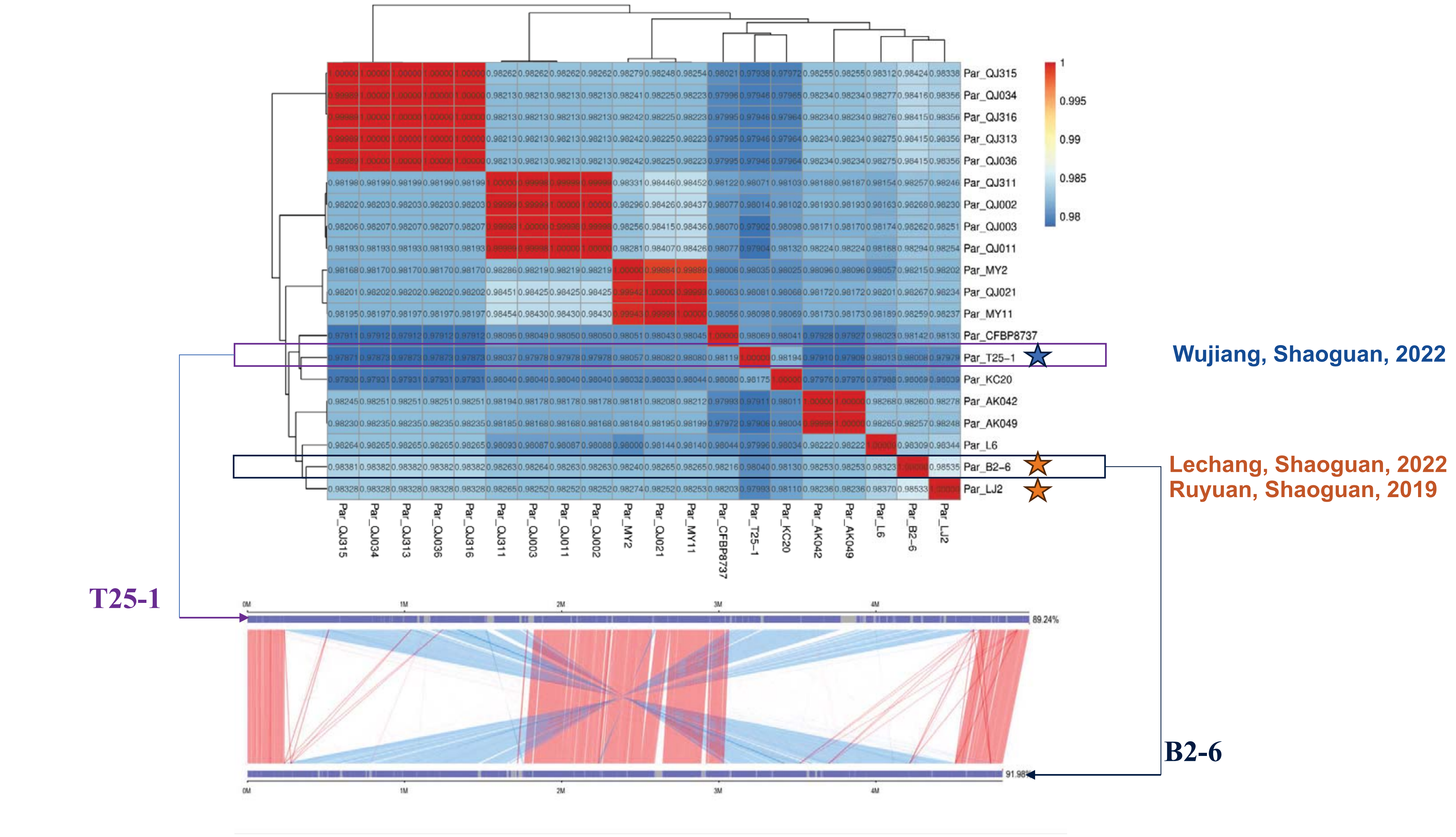

Par_QJ315
Par_QJ034
Par_QJ316
Par_QJ313
Par_QJ036
Par_QJ311
Par_QJ002
Par_QJ003
Par_QJ011
Par_MY2
Par_QJ021
Par_MY11
Par_CFBP8737
Par_T25−1
Par_KC20
Par_AK042
Par_AK049
Par_L6
Par_B2−6
Par_LJ2
Par_QJ315
Par_QJ034
Par_QJ313
Par_QJ036
Par_QJ316
Par_QJ311
Par_QJ003
Par_QJ011
Par_QJ002
Par_MY2
Par_QJ021
Par_MY11
Par_CFBP8737
Par_T25−1
Par_KC20
Par_AK042
Par_AK049
Par_L6
Par_B2−6
Par_LJ2
1
0.995
0.99
0.985
0.98
Wujiang, Shaoguan, 2022
Lechang, Shaoguan, 2022
Ruyuan, Shaoguan, 2019
T25-1
B2-6
0M
1M
2M
3M
4M
89.24%
91.98%

Supplementary Fig. 4
Synteny alignments between
D. fangzhongdai J21-2 and
each typical strain from orchid
and pear trees.

Ochids strains aligned to typical taro strain J21-2

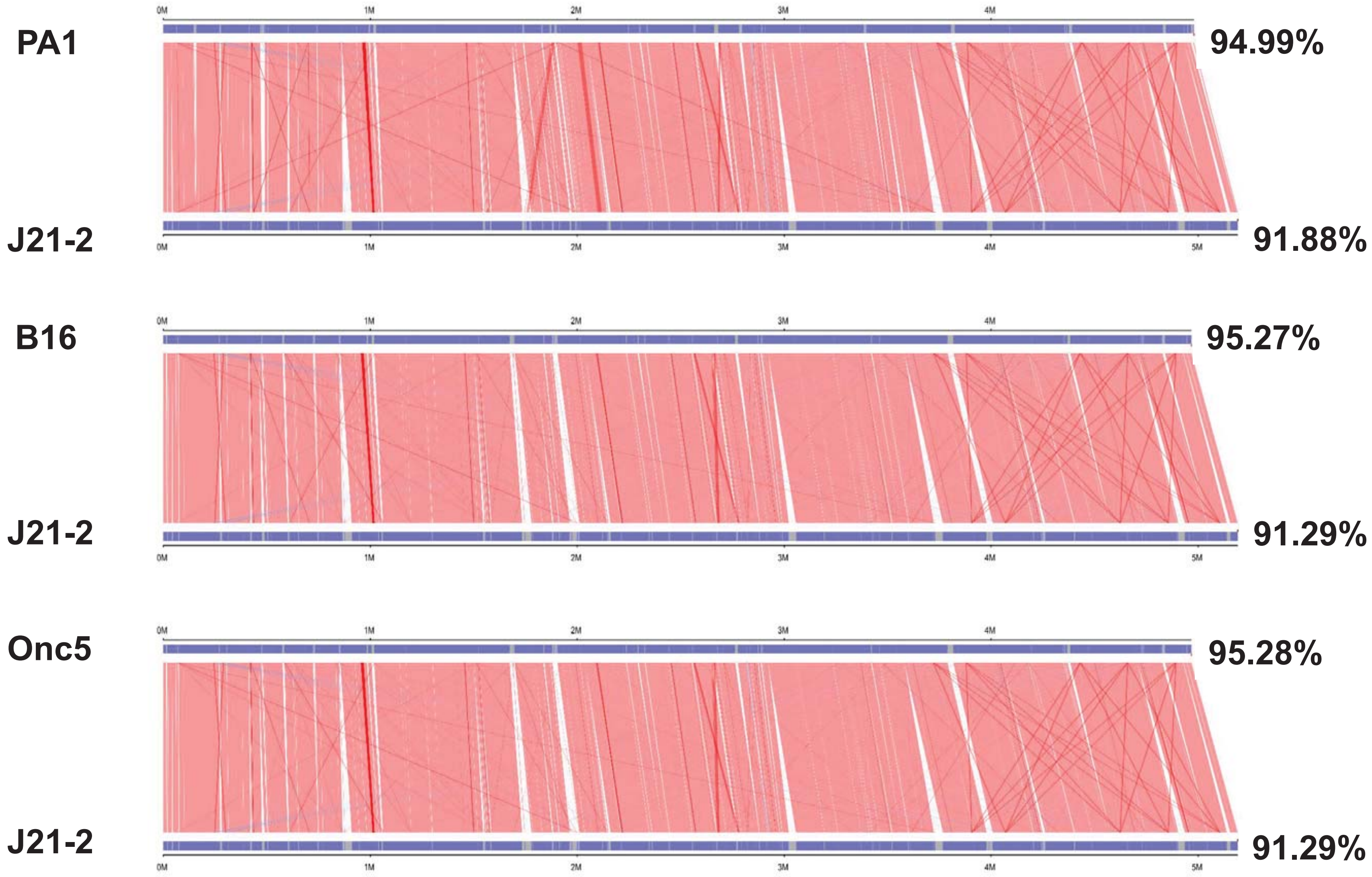

Pear strains aligned to typical taro strain J21-2

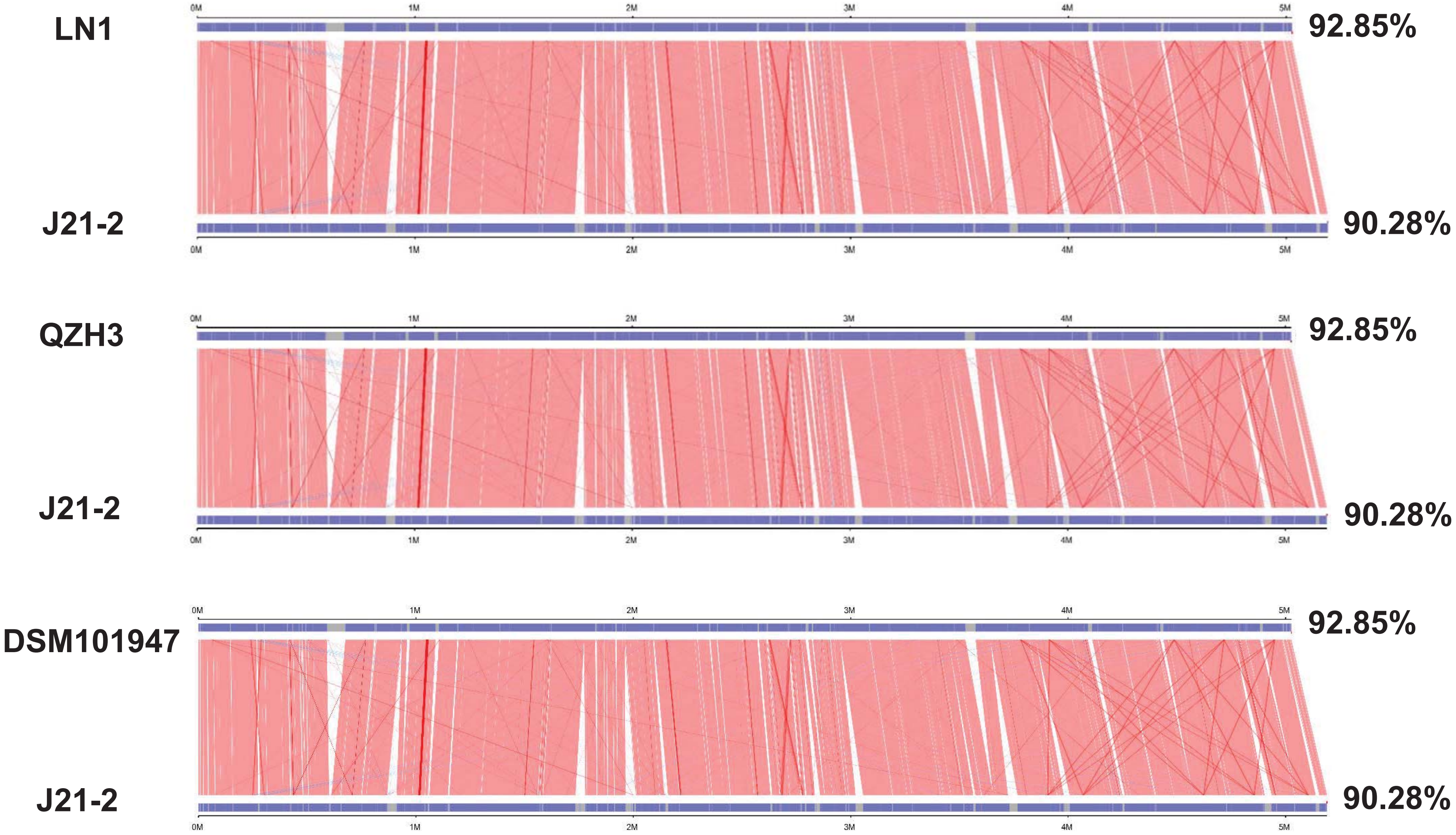

Supplementary Figure 5
Specific genes of each *D. fangzhongdai* strain and their core and pangenome analyses.

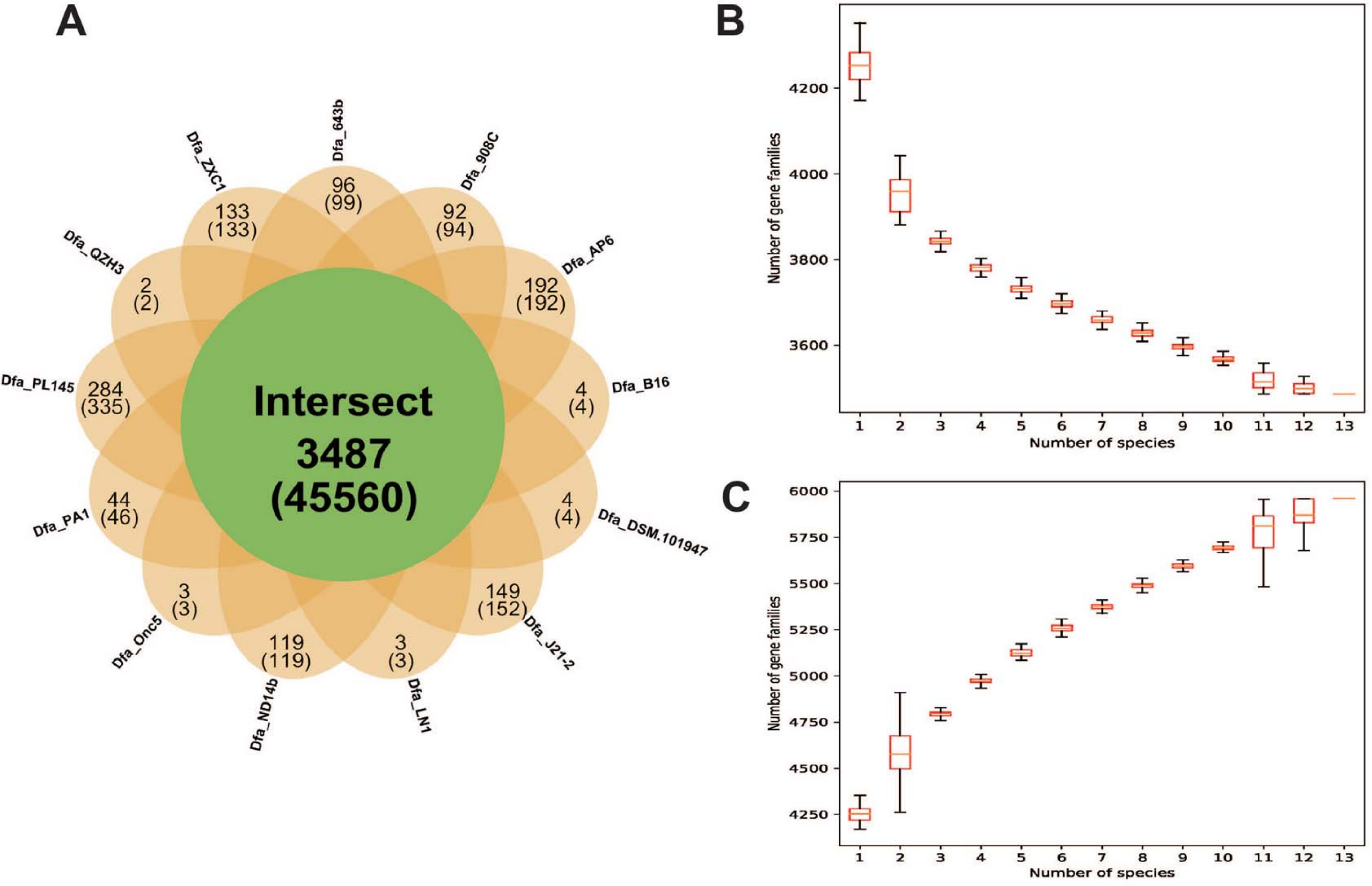
A
Dfa_643b
96
(99)
Dfa_908C
92
(94)
Dfa_AP6
192
(192)
Dfa_B16
4
(4)
Dfa_DSM.101947
4
(4)
Dfa_J21-2
149
(152)
Dfa_LN1
3
(3)
Dfa_ND14b
119
(119)
Dfa_Onc5
3
(3)
Dfa_PA1
44
(46)
Dfa_PL145
284
(335)
Dfa_QZH3
2
(2)
Dfa_ZXC1
133
(133)
Intersect
3487
(45560)
B
Number of gene families
4200
4000
3800
3600
Number of species
1 2 3 4 5 6 7 8 9 10 11 12 13
C
Number of gene families
6000
5750
5500
5250
5000
4750
4500
4250
Number of species
1 2 3 4 5 6 7 8 9 10 11 12 13

Supplementary Figure 6
Average maximum temperature (Tmax) and average minimum temperature (Tmin) value in May from 2011 to 2022 in Shaoguan city, Guangdong Province.

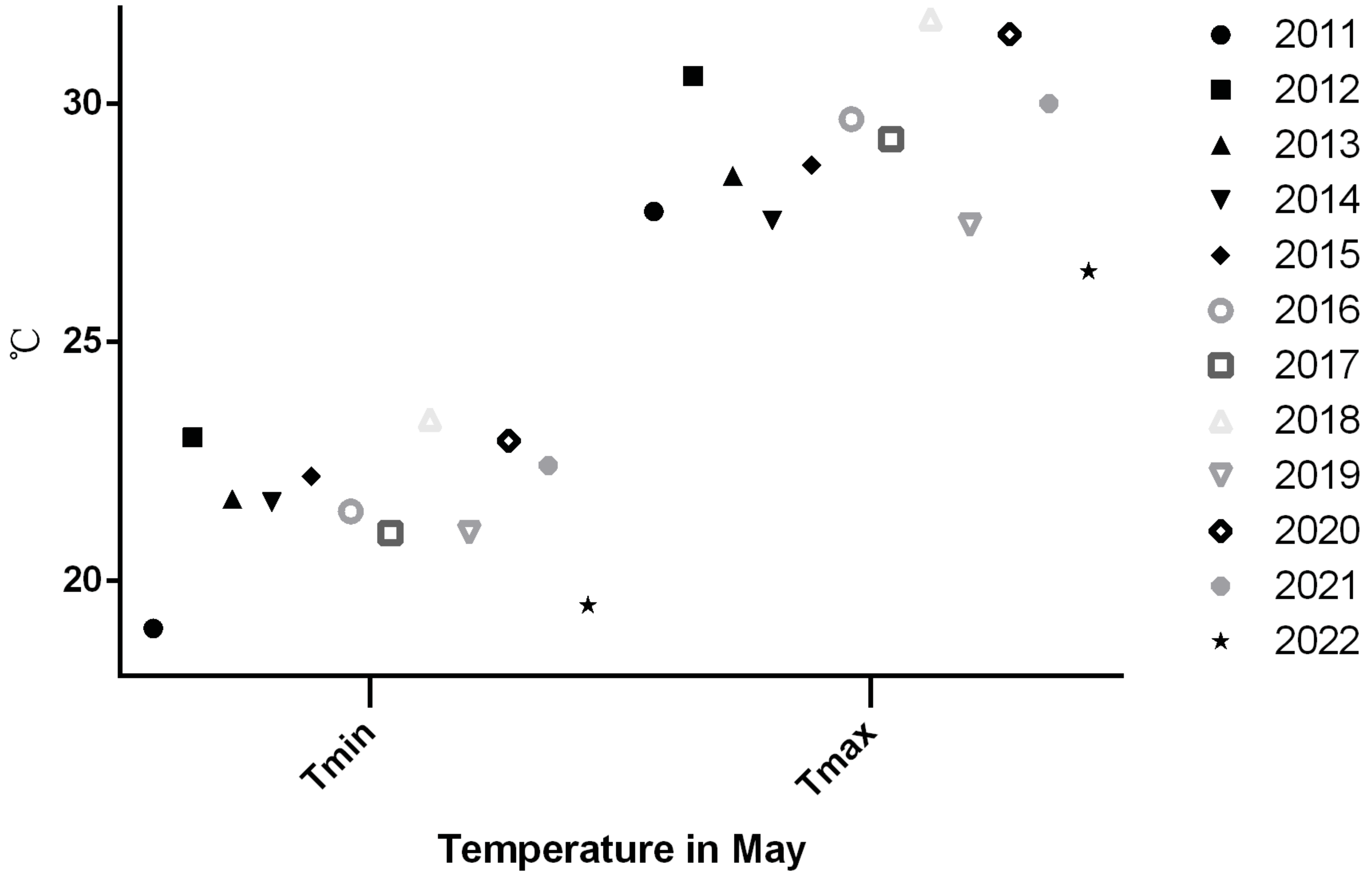

℃
30
25
20
Tmin
Tmax
Temperature in May
2011
2012
2013
2014
2015
2016
2017
2018
2019
2020
2021
2022
